# Cause-specific excess mortality in Denmark, Finland, Norway, and Sweden during the COVID-19 pandemic 2020–2022: a study using nationwide population data

**DOI:** 10.1007/s10654-024-01154-0

**Published:** 2024-09-16

**Authors:** Anton Nilsson, Louise Emilsson, Kasper P. Kepp, Ann Kristin Skrindo Knudsen, Ingeborg Forthun, Christian Madsen, Jonas Björk, Tea Lallukka

**Affiliations:** 1 Epidemiology, Population Studies and Infrastructures (EPI@LUND), Division of Occupational and Environmental Medicine, Lund University, Lund, Sweden; 2 General Practice Research Unit (AFE) and Department of General Practice, Institute of Health and Society, University of Oslo, Oslo, Norway; 3 Vårdcentralen Värmlands Nysäter and Centre for Clinical Research, County Council of Värmland, Värmland, Sweden; 4 Department of Medical Epidemiology and Biostatistics, Karolinska Institute, Solna, Sweden; 5 Section of Biophysical and Biomedicinal Chemistry, Technical University of Denmark, Copenhagen, Denmark; 6 Epistudia, Bern, Switzerland; 7 Department of Disease Burden, Norwegian Institute of Public Health, Bergen, Norway; 8 Clinical Studies Sweden, Forum South, Skåne University Hospital, Lund, Sweden; 9 Department of Public Health, University of Helsinki, Helsinki, Finland

**Keywords:** Pandemic, COVID-19, Cause-specific excess mortality, Nordic countries

## Abstract

While there is substantial evidence on excess mortality in the first two years of the COVID-19 pandemic, no study has conducted a cause-specific analysis of excess mortality for the whole period 2020–2022 across multiple countries. We examined cause-specific excess mortality during 2020–2022 in Denmark, Finland, Norway, and Sweden—four countries with similar demographics and welfare provisions, which implemented different pandemic response policies. To this end, we utilized nationwide register-based information on annual cause-specific deaths stratified by age and sex, and applied linear regression models to predict mortality in 2020–2022 based on the reference period 2010–2019. Excess deaths were obtained by contrasting actual and expected deaths. Additional analyses employed standardization to a common population, as well as population adjustments to account for previous deaths. Our results showed that, besides deaths due to COVID-19 (a total of 32,491 during 2020–2022), all countries experienced excess deaths due to cardiovascular diseases (in total 11,610 excess deaths), and under-mortality due to respiratory diseases other than COVID-19 (in total 9878) and dementia (in total 8721). The excess mortality due to cardiovascular diseases was particularly pronounced in Finland and Norway in 2022, and the under-mortality due to dementia was particularly pronounced in Sweden in 2021–2022. In conclusion, while COVID-19 deaths emerge as the most apparent consequence of the pandemic, our findings suggest that mortality has also been influenced by substitutions between different causes of death and over time, as well as indirect consequences of COVID-19 infection and pandemic responses—albeit to different extents in the different countries.

**Supplementary Information:**

The online version contains supplementary material available at 10.1007/s10654-024-01154-0.

## Introduction

The COVID-19 pandemic has been one of the most severe public health crises of recent times. Reliably assessing the mortality burden of the pandemic is challenging, however, due to potential substitution between different causes of death, incomplete testing and reporting, and a range of possible indirect mortality consequences of the pandemic and pandemic responses. Because of these issues, excess mortality is often considered a better measure of pandemic mortality impact than confirmed and reported COVID-19 deaths [1–3].

Many previous studies have estimated all-cause excess mortality during the COVID-19 pandemic [2–11]. Globally, the first two years of the pandemic, 2020–2021, were estimated to have caused 15–18 million excess deaths [7, 8]. The Nordic countries appear to have experienced lower excess mortality during the pandemic than most countries in Europe and the rest of the world, however with several noticeable differences across the countries and over time [5, 6, 9, 11]. In previous work, we documented that excess mortality in Sweden mainly occurred in 2020, whereas in the other major Nordic countries—Denmark, Finland, and Norway—it mainly took place in 2022 [11]. These striking differences in the timing of pandemic impact may reflect differences in pandemic mitigation policies, where the restrictions imposed in Sweden were fewer, typically less binding, and in several cases implemented later than those in the other Nordic countries [12–14]. Notably, while the total number of confirmed deaths due to COVID-19 per million persons during the 2020–2022 period was higher in Sweden than in the other Nordic countries [15], Sweden stood out as one of the Nordic countries with the lowest rates of all-cause excess mortality over the same period of time [11]. This pattern raises questions about the pandemic response measures and other factors that may have differed across the Nordics, and what causes of death may have developed more favorably in Sweden than in the other countries.

Evidence on cause-specific excess mortality during the COVID-19 pandemic has been documented for several countries across the globe [16–27]. Overall, the evidence has pointed at excess mortality due to cardiovascular diseases [16, 17, 19, 20, 22–25, 27] and diabetes [16, 17, 19, 22, 23, 26], as well as in Alzheimer’s/dementia (at least in the beginning of the pandemic) [17, 23, 24], and under-mortality due to non-COVID respiratory diseases [18, 21, 23, 24, 27]. For other deaths, such as due to cancer, evidence has generally suggested little or no impact [18, 19, 22–25].

Since data on causes of death require information from doctor’s certificates and classification by statistical offices, such data are only available with a delay, often by up to two years for confirmed cases. As a result, previous studies on cause-specific excess mortality during the COVID-19 pandemic have so far mainly been conducted for 2020 and 2021. Moreover, there is little evidence from the Nordic countries on cause-specific mortality during the pandemic. For Sweden in 2020, one study documented an under-mortality due to cardiovascular disease, as well as an under-mortality due to cancer [4]. However, this analysis did not account for time trends in mortality, which can have substantial impacts on results [6]. A study on Norwegian data from 2020–2022 documented an excess mortality due to cardiovascular disease and malignant tumors, as well as an under-mortality due to non-COVID-19 respiratory diseases and dementia [28]. More comprehensive evidence on cause-specific excess mortality in the Nordics in 2020–2022 is currently lacking, however, an issue that hinders more general conclusions on cause-specific impacts and how these may have varied across countries with different pandemic response policies.

In this study, we utilized annual death counts from population registers to examine cause-specific excess mortality in the Nordic countries Denmark, Finland, Norway, and Sweden during 2020–2022. Notably, these four countries are all similar with respect to population structure, culture, and welfare provisions, but implemented different response policies during the COVID-19 pandemic, with Sweden arguably being the clearest outlier. We assessed differences and similarities in patterns of cause-specific mortality across the four countries and the three pandemic years.

## Data and methods

### Data

Each Nordic country has a government agency responsible for population health statistics, which collects death certificates of deceased individuals. The death certificate contains information on underlying, intermediate, immediate, and contributing causes of death, and is filled out by a certifying physician, who sends the certificate to the agency either electronically or by mail. Denmark introduced a fully electronic system for submission of death certificates in 2007 [29], and Norway did so gradually from 2018 to 2022 [28, 30]. In Finland and Sweden, electronic submission and submission by mail are both possible [31, 32].

Death certificates are reviewed by the responsible agency and are processed by computer applications such as the Iris software [33]. The review aims to ensure that causes of death are coded consistently with the guidelines by the World Health Organization (WHO), and may result in the underlying cause of death to be replaced by a different one. Once an individual’s causes of death have been finalized, a record is entered into the country’s Cause of Death Register. Based on this register, the agency publishes annual age- and sex-aggregated data on deaths by underlying causes.

For this study, we obtained the age- and sex-aggregated data on underlying causes of deaths from the Cause of Death Registers in Denmark, Finland, Norway, and Sweden for the years 2010–2022. Data on population sizes for the corresponding years were obtained from the *Nordic Statistics database*, a website that collects data from the statistical agencies in the Nordic countries. (See the Data Availability statement for more details.)

For Denmark, Finland, and Sweden, deaths were reported in five-year age intervals (0–4, 5–9, 10–14, 15–19, etc.), with a few exceptions. In particular, in Finland, the 0–4-year group had been split into 0-year-olds and 1–4-year-olds. Moreover, in Denmark everyone aged 85 and older had been combined into one group, and in Finland and Sweden, everyone aged 95 of older had been combined into one group. For Norway, deaths were reported in ten-year-intervals, except that 0–19-year-olds had been combined into one group, as well as those aged 90 and older, yielding the age intervals 0–19, 20–29, 30–39, 40–49, 50–59, 60–69, 70–79, 80–89 and ≥ 90. The data on population sizes by age and sex were obtained in one-year age groups, which for each country we combined to form the same age intervals as for which deaths were reported.

For Denmark, both total and strata-specific death counts had been rounded to the nearest integer ending with 0 or 5. Moreover, for Denmark and Norway, strata-specific death counts were sometimes reported as missing; this occurred when the actual counts were low (counts of 1–4 in Denmark; 0–3 in Norway). In the reported analysis, we replaced such values with their average possible value (2.5 in Denmark; 1.5 in Norway). Instead replacing these values by their largest or smallest possible value made negligible differences to the results.

We examined the following major causes of death: COVID-19, respiratory diseases other than COVID-19, cardiovascular diseases, cancer, diabetes, dementia, and external causes of death. To avoid undercounting of cancers, we included not only malignant but also benign tumors in the cancer category (the latter made up 0–3% of all tumor deaths across countries). A category of “other” deaths was also created; this included all deaths not part of the listed categories. Supplementary Table S1 shows how the causes of death were defined in terms of ICD-10 codes. Overall, the ICD-10 codes defining the groups were the same for all countries. The main exception was observed for Finland, where external causes of deaths did not include alcohol-related accidents; the latter were hence instead counted as “other.” Besides cause-specific deaths, we also assessed all-cause deaths, replicating previous investigations [5, 11] as a point of reference.

## Methods

Our method involved the following steps. For each cause of death, country, and age- and sex-stratum, we first:

1. Calculated the mortality rate due to the particular cause for each year 2010–2022 by dividing the number of deaths due to the cause by the mean population size of the stratum in the same year.
2. Applied linear regression of the mortality rate versus year for the years 2010–2019.
3. Used the results from the regressions in step 2 to calculate the expected mortality rate in the stratum for the years 2010–2022.
4. Multiplied each expected mortality rate by the mean population in the corresponding year and stratum to obtain the total number of expected deaths due to a particular cause in that stratum.

After applying these steps, expected deaths were summed up across age- and sex-strata to obtain the total number of expected deaths due to a particular cause in each country and year.

Absolute excess deaths for each country and cause were calculated for the years 2020–2022 as the difference between observed and expected deaths. Standard errors were obtained as the square root of the sum of the squared standard errors of the stratum-specific expected deaths.

We also obtained relative excess deaths by calculating the ratio of observed to expected deaths, by country, cause, and year. Standard errors for relative excess deaths were obtained by the delta method.

For all models, results were reported with 95% prediction intervals.

### Alternative models for trends

In addition to the main analysis using linear trends based on 2010–2019, we applied two alternative methods to model the trends. First, we conducted an analysis using predictions based on the five-year period 2015–2019 only. This analysis could produce more accurate predictions if trends were non-linear. On the other hand, the five-year models would be more sensitive to outliers in particular years during the five year-period. As in the main model, the standard errors of the excess deaths were obtained as the square root of the sum of the squared standard errors of the stratum-specific expected deaths.

Second, we conducted an analysis with trends based on the whole ten-year period 2010–2019, but where we instead of raw mortality used the logarithmic transformation ${\text{ln}}\left( {mortality + 0.00001} \right)$ as the outcome. This model makes the assumption that the effects of time on mortality are multiplicative rather than additive. (Rates were minimally adjusted by 0.00001 to avoid zeros). We refer to this model as the model with log-linear trends. Standard errors of excess deaths were obtained with the delta method.

For each outcome and country, the performances of the three different models were assessed by calculating weighted averages of the adjusted R^2^ obtained within different age- and sex-strata, with weights proportional to the number of individuals belonging to the different strata in year 2020.

### Common population

To allow for cross-country and temporal comparisons of the mortality burden of the pandemic while accounting for variations in age and sex, we conducted a variant of our main analysis where we standardized mortality to deaths per million individuals based on the population distribution of Denmark in 2020. Specifically, we here modified step 4 of the previously outlined procedure, so that instead of using the actual number of people in each population stratum, we used the size of the corresponding stratum in the Danish population in 2020 (with numbers re-scaled to represent persons per million).

### Population adjustment for previous deaths

Since the populations observed in a given year depend on previous excess mortality, we also conducted an analysis based on the counterfactual populations that would potentially have occurred in the absence of the pandemic. For 2020 and earlier, these populations were set equal to the actual, observed populations. For 2021, the observed size of each age- and sex-specific stratum was adjusted by adding the (positive or negative) number of excess deaths estimated to have affected the corresponding group in 2020. Here, an assumption was made that deaths were distributed evenly within the age groups for which excess deaths had been estimated. For example, a counterfactual population of 70–74-year-old males in Sweden in 2021 was obtained by taking the actual population in this group and adding one fifth of the estimated excess deaths among 65–69-year-old males in Sweden in 2020 and four fifths of the estimated excess deaths among 70–74-year-old males in Sweden in 2020. To create counterfactual populations for 2022, we calculated excess deaths in 2021 given the populations obtained in the previous step and added the relevant excess deaths from both 2020 and 2021 to each age- and sex-group in 2022. The main analysis was replicated using these adjusted population sizes. We then calculated the sums of the cause-specific excess deaths across the years 2020–2022 based on this approach, thereby evaluating the total pandemic impact on deaths due to different causes.

## Results

### Descriptives and trends

For most countries, age groups, sexes, and causes of death, visual inspection suggested that mortality developed roughly linearly over the 2010–2019 reference period (see Supplementary Figs. S1–S30 for mortality in each stratum, together with ten-year trend lines). Possible exceptions included “other” deaths in Denmark (where a trend break may have occurred around 2016) and cancer deaths in Sweden (where a trend break might have occurred around 2018). All-cause mortality in Denmark appeared somewhat non-linear as well. The fluctuations around trends varied across outcomes and countries, with particularly large variability seen for respiratory mortality, while smaller for cardiovascular mortality throughout the reference period.

Supplementary Figs. S31–S34 show the observed numbers of deaths by country and cause, together with predictions from the three different models (the main model with linear ten-year trends, as well as the model with linear five-year trends and the model with log-linear ten-year trends). In general, the different models all appeared to fit the data reasonably well.

**Fig. 1:**
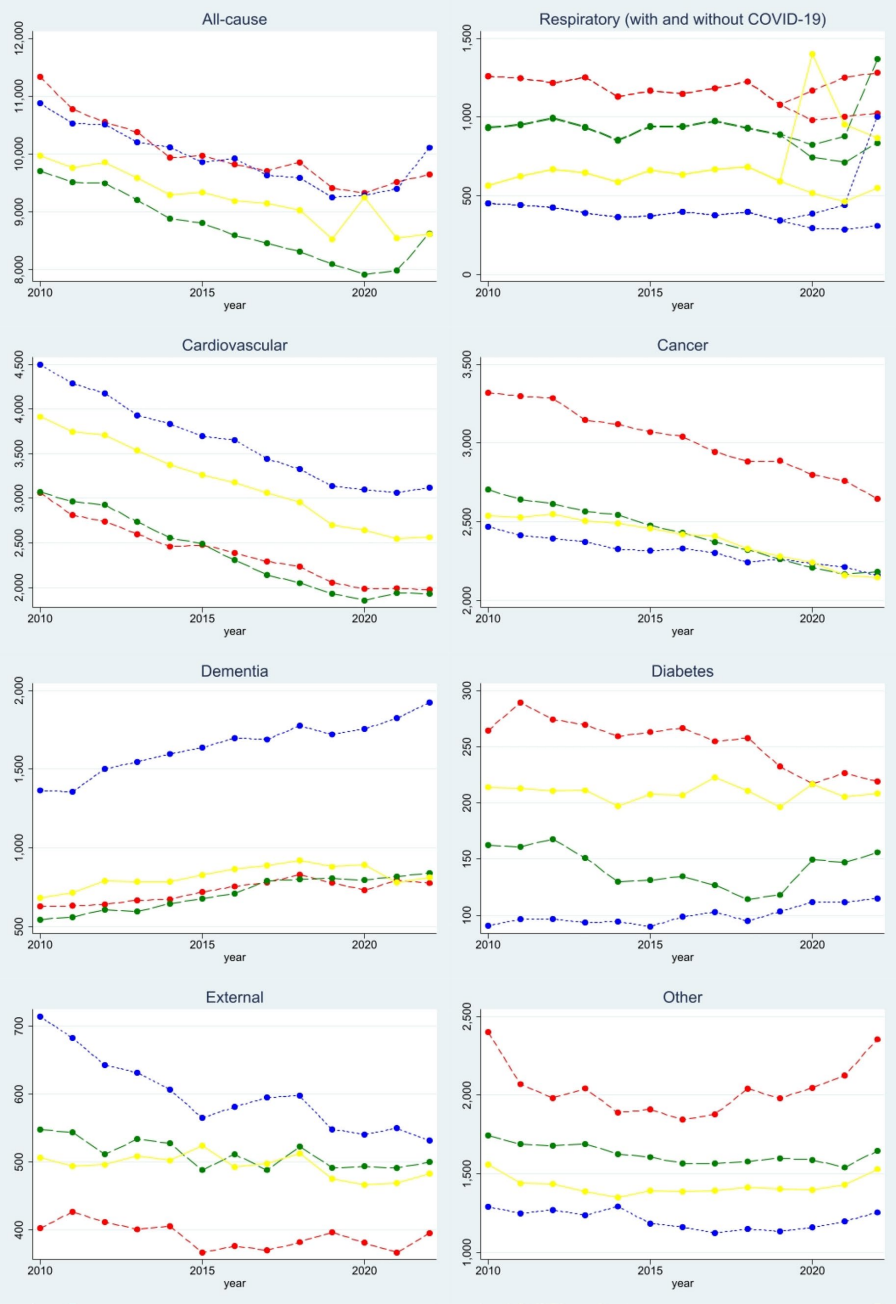
Standardized mortality per million people in Denmark (), Finland (), Norway (), and Sweden (). Note: The reference population is Denmark in 2020

The developments of mortality, standardized to the common population of Denmark in 2020, are shown in Fig. 1. Throughout the time period, Denmark displayed the largest standardized mortality due to non-COVID-19 respiratory diseases, cancer, diabetes, and “other” deaths, but the lowest standardized mortality due to external causes. Finland stood out as the country with the highest standardized mortality due to cardiovascular diseases, dementia, and external causes, but with the lowest due to non-COVID-19 respiratory diseases, cancer, and “other” causes.

### Excess mortality

**Fig. 2:**
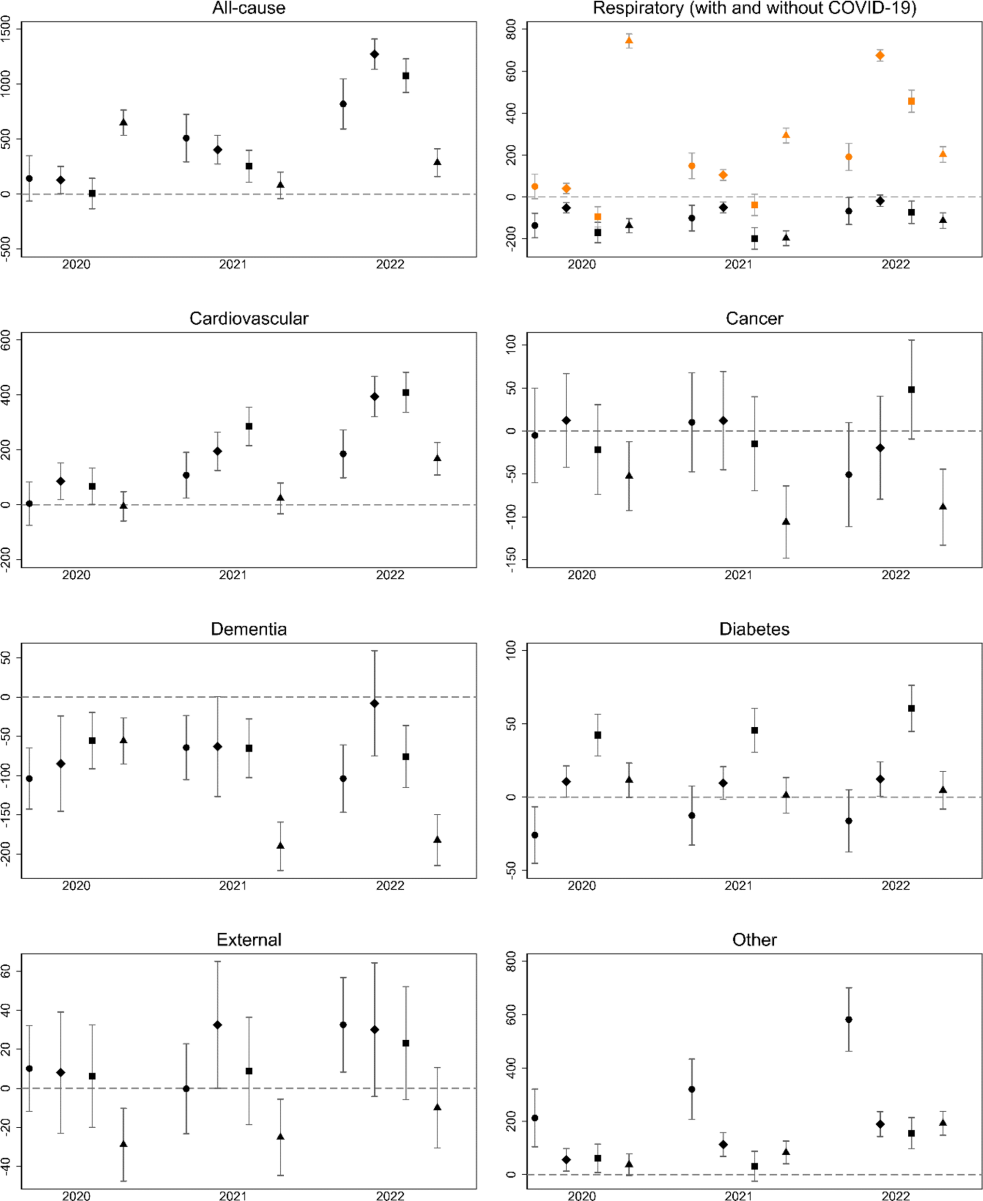
Standardized excess mortality per million people in Denmark (), Finland (), Norway (), and Sweden () in 2020, 2021, and 2022. Notes: The figure shows 95% prediction intervals. The reference population is Denmark in 2020. Excess mortality was calculated based on linear ten-year trends. In the panel with respiratory deaths, the numbers indicated by orange markers include deaths due to COVID-19, whereas those indicated by black markers do not

Our main estimates of excess mortality during 2020–2022, based on linear predictions using the 2010–2019 reference period, are reported in Table 1. Results based on the 2015–2019 reference period are found in Supplementary Table S2, and results from the model with log-linear trends in Supplementary Table S3. The performances (adjusted R^2^) of the different models are shown in Supplementary Table S4. The numbers suggest that the main model may be preferable to the model with five-year trends, whereas it is essentially indistinguishable from the model with log-linear trends. Overall, the estimates from the different models were similar, but some—especially from the models with five-year trends—deviated from the main results. We comment briefly below on cases where results from the model with five-year trends were substantially different from the main results. Excess deaths per million people according to the main model, standardized to the Danish 2020 population, are shown in Fig. 2.

**Table 1:** Excess deaths

Cause	Year	Denmark			Finland			Norway			Sweden		
		Deaths (actual & expected)	Absolute excess deaths (95% PI)	Relative excess deaths (95% PI)	Deaths (actual & expected)	Absolute excess deaths (95% PI)	Relative excess deaths (95% PI)	Deaths (actual & expected)	Absolute excess deaths (95% PI)	Relative excess deaths (95% PI)	Deaths (actual & expected)	Absolute excess deaths (95% PI)	Relative excess deaths (95% PI)
All-cause	2020	54,400; 53,567	833 (− 372; 2038)	1.02(0.99; 1.04)	55,498; 54,802	696 (− 10; 1402)	1.01 (1.00; 1.03)	40,558; 40,590	− 32 (− 751; 687)	1.00 (0.98; 1.02)	98,308; 91,196	7112 (5911; 8313)	1.08 (1.06; 1.09)
	2021	56,870; 53,820	3059 (1749; 4351)	1.06 (1.03; 1.08)	57,632; 55,232	2400 (1641; 3159)	1.04 (1.03; 1.06)	41,713; 40,514	1199 (429; 1969)	1.03 (1.01; 1.05)	92,079; 91,227	852 (− 430; 2134)	1.01 (1.00; 1.02)
	2022	59,120; 54,081	5039 (3632; 6446)	1.09 (1.06; 1.12)	63,172; 55,242	7930 (7119; 8741)	1.14 (1.12; 1.17)	45,947; 40,282	5665 (4844; 6486)	1.14 (1.12; 1.16)	94,823; 91,647	3176 (1794; 4558)	1.03 (1.02; 1.05)
COVID-19	2020	1075; –	1075 (–)	–	558; –	558 (–)	–	414; –	414 (–)	–	9495; –	9495 (–)	–
	2021	1480; –	1480 (–)	–	952; –	952 (–)	–	864; –	864 (–)	–	5321; –	5321 (–)	–
	2022	1590; –	1590 (–)	–	4349; –	4349 (–)	–	2858; –	2858 (–)	–	3535; –	3535 (–)	–
Respiratory (non-COVID-19)	2020	5720; 6515	− 795 (− 1136; − 454)	0.88 (0.83; 0.92)	1749; 2067	− 318 (− 459; − 177)	0.85 (0.79; 0.9)	3768; 4639	− 871 (− 1118; − 624)	0.81 (0.77; 0.86)	5550; 6994	− 1444 (− 1809; − 1079)	0.79 (0.75; 0.83)
	2021	5995; 6616	− 621 (− 988; − 254)	0.91 (0.86; 0.96)	1766; 2077	− 311 (− 466; − 156)	0.85 (0.79; 0.91)	3660; 4731	− 1071 (− 1334; − 808)	0.77 (0.73; 0.82)	5043; 7172	− 2129 (− 2517; − 1741)	0.70 (0.67; 0.74)
	2022	6300; 6719	− 419 (− 817; − 21)	0.94 (0.88; 0.99)	1940; 2077	− 137 (− 304; 30)	0.93 (0.86; 1.01)	4401; 4807	− 406 (− 686; − 126)	0.92 (0.86; 0.97)	6173; 7389	− 1216 (− 1633; − 799)	0.84 (0.79; 0.88)
Cardiovascular	2020	11,560; 11,549	11 (− 452; 474)	1.00 (0.96; 1.04)	18,496; 17,994	502 (116; 888)	1.03 (1.00; 1.05)	9577; 9235	342 (− 3; 687)	1.04 (1.00; 1.08)	28,111; 28,128	− 17 (− 566; 532)	1.00 (0.98; 1.02)
	2021	11,935; 11,277	658 (160; 1156)	1.06 (1.01; 1.11)	18,789; 17,605	1184 (767; 1601)	1.07 (1.04; 1.10)	10,205; 8746	1459 (1091; 1827)	1.17 (1.12; 1.22)	27,464; 27,194	270 (− 314; 854)	1.01 (0.99; 1.03)
	2022	12,130; 10,987	1143 (602; 1684)	1.10 (1.05; 1.16)	19,526; 17,024	2502 (2055; 2949)	1.15 (1.11; 1.19)	10,381; 8192	2189 (1795; 2583)	1.27 (1.21; 1.33)	28,251; 26,354	1897 (1270; 2524)	1.07 (1.05; 1.10)
Cancer	2020	16,315; 16,352	− 37 (− 358; 284)	1.00 (0.98; 1.02)	13,411; 13,338	73 (− 248; 394)	1.01 (0.98; 1.04)	11,144; 11,275	− 131 (− 409; 147)	0.99 (0.96; 1.01)	23,532; 24,092	− 560 (− 974; − 146)	0.98 (0.96; 0.99)
	2021	16,470; 16,405	65 (− 280; 410)	1.00 (0.98; 1.03)	13,550; 13,501	49 (− 296; 394)	1.00 (0.97; 1.04)	11,216; 11,283	− 67 (− 365; 231)	0.99 (0.97; 1.02)	23,070; 24,228	− 1158 (− 1603; − 713)	0.95 (0.93; 0.97)
	2022	16,135; 16,449	− 314 (− 686; 58)	0.98 (0.96; 1.00)	13,495; 13,610	− 115 (− 483; 253)	0.99 (0.95; 1.03)	11,537; 11,269	268 (− 51; 587)	1.02 (0.99; 1.05)	23,436; 24,430	− 994 (− 1474; − 514)	0.96 (0.94; 0.98)
Dementia	2020	4270; 4872	− 602 (− 829; − 375)	0.88 (0.84; 0.92)	10,673; 11,211	− 538 (− 885; − 191)	0.95 (0.92; 0.98)	4067; 4376	− 309 (− 495; − 123)	0.93 (0.89; 0.97)	9709; 10,303	− 594 (− 900; − 288)	0.94 (0.91; 0.97)
	2021	4785; 5151	− 366 (− 611; − 121)	0.93 (0.88; 0.97)	11,455; 11,861	− 406 (− 780; − 32)	0.97 (0.93; 1.00)	4265; 4630	− 365 (− 565; − 165)	0.92 (0.88; 0.96)	8652; 10,747	− 2095 (− 2422; − 1768)	0.81 (0.78; 0.83)
	2022	4805; 5450	− 645 (− 910; − 380)	0.88 (0.84; 0.92)	12,313; 12,388	− 75 (− 475; 325)	0.99 (0.96; 1.03)	4442; 4867	− 425 (− 637; − 213)	0.91 (0.87; 0.95)	9201; 11,285	− 2084 (− 2439; − 1729)	0.82 (0.79; 0.84)
Diabetes	2020	1275; 1416	− 141 (− 253; − 29)	0.90 (0.83; 0.97)	667; 596	71 (10; 132)	1.12 (0.98; 1.26)	762; 545	217 (146; 288)	1.40 (1.22; 1.58)	2316; 2196	120 (− 3; 243)	1.05 (1.00; 1.11)
	2021	1355; 1430	− 75 (− 198; 48)	0.95 (0.87; 1.03)	672; 614	58 (− 9; 125)	1.09 (0.93; 1.26)	787; 527	260 (184; 336)	1.49 (1.28; 1.71)	2235; 2230	5 (− 128; 138)	1.00 (0.94; 1.06)
	2022	1340; 1444	− 104 (− 237; 29)	0.93 (0.84; 1.01)	714; 629	85 (14; 156)	1.14 (0.94; 1.33)	843; 506	337 (255; 419)	1.67 (1.39; 1.94)	2317; 2277	40 (− 103; 183)	1.02 (0.95; 1.08)
External	2020	2188; 2163	24 (− 103; 151)	1.01 (0.95; 1.07)	3084; 3074	10 (− 162; 182)	1.00 (0.93; 1.07)	2638; 2609	29 (− 112; 170)	1.01 (0.96; 1.07)	4871; 5190	− 319 (− 509; − 129)	0.94 (0.90; 0.97)
	2021	2140; 2175	− 35 (− 172; 102)	0.98 (0.92; 1.05)	3221; 3039	182 (− 2; 366)	1.06 (0.97; 1.15)	2657; 2622	35 (− 116; 186)	1.01 (0.96; 1.07)	4969; 5241	− 272 (− 474; − 70)	0.95 (0.91; 0.98)
	2022	2348; 2191	156 (9; 303)	1.07 (1.00; 1.14)	3147; 2994	153 (− 43; 349)	1.05 (0.95; 1.16)	2747; 2630	117 (− 42; 276)	1.04 (0.98; 1.11)	5196; 5312	− 116 (− 332; 100)	0.98 (0.94; 1.02)
Other	2020	11,998; 10,700	1298 (667; 1929)	1.12 (1.06; 1.19)	6860; 6523	337 (94; 580)	1.05 (1.01; 1.09)	8188; 7884	304 (28; 580)	1.04 (1.00; 1.07)	14,724; 14,293	431 (12; 850)	1.03 (1.00; 1.06)
	2021	12,710; 10,766	1944 (1264; 2624)	1.18 (1.11; 1.26)	7227; 6535	692 (431; 953)	1.11 (1.06; 1.16)	8059; 7948	111 (− 183; 405)	1.01 (0.98; 1.05)	15,325; 14,415	910 (461; 1359)	1.06 (1.03; 1.10)
	2022	14,473; 10,840	3632 (2897; 4367)	1.34 (1.24; 1.43)	7688; 6520	1168 (888; 1448)	1.18 (1.12; 1.24)	8738; 7985	753 (437; 1069)	1.09 (1.05; 1.14)	16,714; 14,600	2114 (1632; 2596)	1.14 (1.11; 1.18)

Expected deaths were estimated based on strata-specific ten-year trends (2010–2019). Prediction intervals (PIs) were obtained as estimates ± 1.96*standard error

All-cause excess mortality per standardized million people was particularly high in Sweden in 2020 and in Denmark, Finland, and Norway in 2022. For Sweden in 2020, the all-cause deaths were 8% higher than the expected ones. For Denmark, Finland, and Norway in 2022, the all-cause death counts exceeded the expected ones by 9–14% (or 5% in Denmark according to the model with five-year trends).

Overall, COVID-19 was the most important contributor to excess mortality. Countries and years with larger numbers of COVID-19 deaths tended to coincide with countries and years with high excess mortality. The main exception was Sweden in 2021, for which a low all-cause mortality was seen, despite the relatively large number of COVID-deaths.

For all countries and years during the 2020–2022 period, there was an under-mortality due to non-COVID-19 respiratory diseases. In some case (for Norway 2020–2021), this under-mortality even outweighed the number of deaths due to COVID-19. The relative under-mortality due to non-COVID-19 respiratory diseases ranged between 6 and 30% across countries and years. Overall, Sweden and Norway were the countries with the largest under-mortality due to non-COVID-19 respiratory diseases per standardized million people. This pattern was particularly clear in 2021, with statistically significant differences compared to Denmark and Finland.

Overall, an excess mortality due to cardiovascular diseases was observed, although for some countries this only reached statistical significance in the last one or two years of the study period. The relative excess deaths ranged between 0 and 27%, with particularly large numbers for Finland and Norway, and for 2022 in general.

Cancer deaths were roughly within the expected ranges for Denmark, Finland, and Norway throughout the period 2020–2022. In Sweden, there was an under-mortality due to cancers. However, the graphical evidence (Supplementary Fig. S34) indicated a trend break in cancer deaths in Sweden already before the pandemic, and according to the model with five-year trends, cancer mortality was within the expected range in Sweden as well.

An under-mortality in deaths classified as dementia was observed across all countries and for all years but was particularly pronounced for Sweden in 2021 and 2022. For Finland, the under-mortality in dementia deaths was small and non-significant in 2022, and the results for this country were further sensitive to the choice of trends. The model based on five-year trends instead suggested an excess mortality in dementia for Finland in 2022, and no clear deviations from the trend in 2020–2021.

For diabetes, patterns were mixed, but with no evidence of strong of impacts in Denmark, Finland, or Sweden. For Norway, however, a substantial excess mortality due to diabetes was seen throughout the three-year period.

Deaths due to external causes were generally within the expected ranges, except for Sweden, which appeared to experience an under-mortality due to external causes in the beginning of the pandemic (although not significantly so when applying five-year trends), and Denmark, which appeared to experience an excess mortality due to external causes in the end of the period (although not when applying five-year trends). Deaths due to “other” conditions were elevated in all countries, especially so in Denmark (these results were however moderated in analyses based on five-year trends).

### Population adjustment

Supplementary Table S5 shows results based on analyses where populations were adjusted for previous excess deaths, hence better reflecting a counterfactual scenario where the pandemic had never occurred. For Denmark, Norway, and Finland, results were very similar to those without population adjustment, reflecting the low excess mortality in 2020–2021. For Sweden, the population correction led to an increase in the expected numbers of deaths during 2021 and 2022 by between 0.5 and 1.2%, depending on the cause. This somewhat reduced the number of excess deaths in 2021 and 2022 (in particular, all-cause deaths reduced from 852 to 37 in 2021 and from 3171 to 2594 in 2022). Summing up the excess deaths across the three years, we find that the pandemic may have caused a total of 8757 excess deaths in Denmark, 10,978 in Finland, 6882 in Norway, and 9743 in Sweden. These numbers correspond to 0.15%, 0.20%, 0.13%, and 0.09% of the countries’ population sizes in 2020, respectively.

In Denmark, Finland, and Norway, COVID-19 deaths corresponded to roughly half of the estimated all-cause excess deaths over the three years. In Sweden, in contrast, COVID-19 deaths were almost twice as many as the estimated all-cause excess deaths. The result for Sweden reflected this country’s large under-mortalities in dementia and cancer, and a comparatively small excess mortality due to cardiovascular disease. Across the four countries combined, we find that, beyond a cumulative count of 32,491 COVID-19 deaths, the pandemic may have resulted in an additional 11,610 cardiovascular deaths, 9878 fewer non-COVID-19 respiratory deaths, and 8721 fewer dementia deaths during 2020–2022.

## Discussion

In this study, we estimated cause-specific excess mortality in the four largest Nordic countries Denmark, Finland, Norway, and Sweden during the COVID-19 pandemic 2020–2022. During this period, all-cause mortality—as well as deaths attributable to COVID-19—was particularly large in Sweden in 2020, and in Denmark, Finland, and Norway in 2022. We here added several pieces of evidence which allowed us to shed light on the developments in the Nordics during this period. First, we observed a substantial under-mortality due to non-COVID-19 respiratory diseases and dementia that occurred across the four countries during the pandemic, not least for Sweden. Second, a substantial excess mortality due to cardiovascular disease was observed across countries and time, especially so for Finland and Norway, and for all countries towards the end of the period. Third, deaths due to cancer, diabetes, and external causes developed roughly in line with their pre-pandemic trends. A few exceptions to this pattern could be noted, including a steep increase in deaths due to diabetes in Norway and an under-mortality in deaths due to cancer in Sweden (the latter only in the models with ten-year trends).

Most major patterns that we have documented were qualitatively similar across the countries, but the differences in timing and magnitude were noticeable. In terms of deaths attributable to COVID-19, Sweden’s comparatively high mortality in 2020 may largely reflect this country’s relatively lenient restrictions. The 2022 surge in COVID-19 deaths in Denmark, Norway, and Finland might be largely related to the emergence of the more contagious Omicron variant, coupled with the easing of restrictions in the beginning of 2022. Sweden’s comparatively low mortality from COVID-19 in this year may reflect the strong pandemic impact in this country in 2020, which may have given rise to a mortality displacement over time, as well as a greater infection-induced immunity. Moreover, Sweden entered 2022 with a higher coverage of the booster vaccine dose than the other countries [34], further contributing to higher immunity. In total across the three pandemic years, Sweden experienced the largest number of COVID-19 deaths among the Nordic countries, even when considering these numbers in relation to population size. At the same time, Sweden had the smallest estimated all-cause excess mortality in relation to its population size when summing deaths over the three years. This comparatively small all-cause excess mortality reflects a larger under-mortality in dementia, a comparatively small excess mortality in cardiovascular deaths, and a possible under-mortality in cancer deaths in Sweden.

The under-mortality we observed in non-COVID-19 respiratory diseases for all the Nordic countries is in line with previous evidence from several countries including South Korea, the US, Australia, and several European countries [18, 21, 23–25, 27], and most likely reflects a reduced spread of infectious diseases, due to social distancing [35]. In addition, pathogenic competition may have played a role; becoming infected by COVID-19 might reduce the risk of contracting other respiratory infections, reducing the spread of non-COVID-19 respiratory diseases [36, 37]. The latter may be one reason why also Sweden, despite its less stringent restrictions, experienced substantial reductions in non-COVID-19 respiratory deaths.

The observed under-mortality in dementia is at odds with previous evidence from the US and Italy, which has instead suggested an excess mortality in dementia during the COVID-19 pandemic [17, 22, 23, 38]. Evidence from the UK suggested that there was an initial excess mortality in dementia, which was however followed by an under-mortality due to the same cause [24]. One likely explanation for the divergent results is that reporting of dementia deaths is subject to substantial measurement error [39]. For example, several autopsy studies have shown that deaths classified as being due to dementia often represent deaths due to respiratory disease such as pneumonia [40–43]. In line with this, a reduction in non-COVID-19 respiratory disease due to social distancing or other factors may have contributed to a reduction in deaths classified as dementia in the Nordic countries (while adherence to WHO guidelines for reporting of COVID-19 deaths [44], along with substantial testing for COVID-19, should have limited the likelihood of COVID-19 deaths being attributed to dementia).

Furthermore, those who died from COVID-19 were, at least in the early stages of the pandemic in both the Nordics and elsewhere, largely made up of older people residing in nursing homes [45–47]. This population has a very high prevalence of dementia [48, 49]. Consequently, the pandemic may also have caused a displacement from dementia deaths to COVID-19 deaths. While COVID-19 deaths peaked in Sweden in 2020, dementia deaths were primarily lowered in 2021 and 2022. This might reflect that many frail individuals with dementia died prematurely in 2020, hence reducing the population of individuals with dementia who would be at risk of dying in 2021 and 2022. Future work should assess how long-lasting this under-mortality in deaths classified as dementia will turn out to be, and whether similar patterns will be detected for Denmark, Norway, and Finland past their peak pandemic year in 2022.

The excess mortality due to cardiovascular disease which we observed for all countries is in line with evidence from several other contexts, including the US and several European countries [16, 17, 19, 20, 22–24, 27, 50–56], although evidence to the contrary exists as well [4, 16, 27, 55]. Several explanations for the observed patterns are possible. First, COVID-19 infection may itself be a risk factor for cardiovascular disease [57–59]. Moreover, healthcare visits for cardiovascular disease have in several countries been found to have decreased during the pandemic [12, 55, 60], a pattern that may represent a hesitancy to seek care, as well as a lower access to care [61, 62]. Worse prevention and management of cardiovascular disease due to disruption of healthcare services during the COVID-19 pandemic has also been documented [63]. More research will be needed to examine how such reductions in the quantity or quality of healthcare visits for cardiovascular disease may have contributed to increased cardiovascular mortality in the Nordic countries. Further examinations will also be necessary to assess the possible impact of other pandemic-related factors on cardiovascular mortality, such as social [64] and physical [65] inactivity, resulting from social distancing.

It is noticeable that excess mortality due to cardiovascular disease in Finland and Norway spiked already before these countries experienced any substantial numbers of COVID-19 deaths. Moreover, Sweden saw comparatively little excess mortality due to cardiovascular disease throughout the three years, and none in 2020, when the country was the most impacted by COVID-19. These observations suggest that factors other than COVID-19 infection, such as restrictions to access to care or hesitancy to seek care, may have been more important contributors to the excess cardiovascular mortality.

Several previous studies have found evidence of excess mortality due to diabetes during the COVID-19 pandemic [16, 17, 19, 22–24, 26, 66], although some countries were also found to exhibit under-mortality due to this cause [16]. Among the previous studies, one reported estimates for Denmark and Finland in 2020 [16]. Our estimates resemble these, but suggest that overall, during 2020–2022, the pandemic had little impact on mortality due to diabetes in Denmark, Finland, and Sweden. For Norway, however, an excess mortality due to diabetes was obtained – one that was much larger than what has been observed in most other contexts. (The finding might be an artifact of changes in the recording of deaths; see the discussion of limitations further down.)

Our results for cancer mortality, which showed no evidence of an impact, at least not in Denmark, Finland, and Norway, are in line with previous literature, which quite consistently has found no evidence of impacts of the COVID-19 pandemic on mortality due to cancer [16, 18, 19, 21–25]. Similarly for deaths due to external causes, our results confirm previous literature which generally suggests impacts to be small or nonexistent [18, 21–24] (a notable exception being deaths due to drug use [23]).

Our study has both strengths and limitations. As for the strengths, the Nordic register data are some of the most complete and accurate in the world [67], making our investigation an ideal case study for the complex mortality dynamics of the pandemic. Our approach accounts for time trends in mortality as well as differences in population age and sex structure over time and across countries—issues that, if unaccounted for, could substantially bias results in investigations of excess mortality [5, 6]. The addition of 2022 means that we covered the post-initial phases of the vaccination programs with the roll-out of booster doses, as well as the increased transmission of COVID-19 also among the vaccinated due to emergence of the Omicron variant.

Among the limitations of our study, the reporting of causes in death certificates comes with a degree of uncertainty, and practices for how deaths are assessed and reported differ across physicians and countries, as well as over time. Indeed, Fig. 1 showed substantial differences in cause-specific mortality before the pandemic, and some of these differences may reflect coding differences. Such coding differences may also have impacted the observed development during the pandemic. As one notable example, deaths classified as dementia are particularly prevalent in Finland, and it is possible that the development of excess mortality due to this cause is not fully comparable across Finland and the other countries.

Norway gradually moved to an electronic system for reporting causes of death during the pandemic years, an issue that might have influenced reporting in several ways, for example by nudging doctors to pick diagnoses that appeared in the predefined dropdown menu [28, 30]. In addition, this change involved an expansion of the maximum number of causes of deaths that were allowed to precede the immediate cause from two to three, meaning that causes that were previously considered too indirect to be included in the death certificate may now be reported as the underlying cause. This could potentially provide an explanation for the substantial increase in diabetes deaths recorded in Norway. However, pandemic impacts on healthcare utilization have also been forward as a possible explanation for the large number of recorded diabetes deaths in this country [30]. Future studies on excess mortality should try to shed more light on the role of coding differences across countries and over time, for example by utilizing data on multiple causes of death.

The rates of testing for COVID-19 varied across the Nordic countries, with Denmark conducting several times more tests per million people than the other countries [15]. This could potentially have led to a greater misclassification of COVID-19 deaths in the other three countries. It is important to note, however, that testing of hospitalized patients was strongly recommended across all countries [68].

Previous evidence has suggested that excess mortality estimates tend to be sensitive to the choice of reference period and other analytical choices [6, 69, 70]. Considering and comparing results from several models is therefore important. In this work, we focused on linear regression models, using a main model that assumed linear time trends over the ten-year period 2010–2019, and extrapolating the estimates to the pandemic years 2020–2022. As deaths are fundamentally recorded as counts, a count data model such as Poisson regression is in principle more adequate than linear regression. One disadvantage of count data models, however, is that there exists no gold standard for the calculation of prediction intervals [71]. A linear regression model offers a convenient way of calculating prediction intervals, and is useful in settings like ours, where populations are large, meaning that mortality rates can be seen as close to continuous. Our use of linear regression models is in line with a number of previous studies of excess mortality [70, 72–78], including our own investigations of all-cause excess mortality in the Nordics during the COVID-19 pandemic [5, 11]. The linear regression model flexibly accommodates non-linear trends, either by restriction of the study period to a shorter time frame (during which non-linearities may be negligible) or by transforming the outcome, such as by taking the logarithm of it, yielding a model with log-linear trends. Notably, the functional form of the linear regression model with log-linear trends resembles that of a Poisson regression model. As an additional check, we have applied Poisson regressions models (Supplementary Table S6). The results confirmed that the excess death estimates from Poisson regression were similar to those from linear regression with log-linear trends.

Compared to the results from our main linear regression model with ten-year trends, the model with five-year trends generally produced lower estimates of excess deaths for Denmark, Finland, and Norway, while larger for Sweden. In particular, applying the model with five-year trends resulted in a reduction of “Other” excess deaths in Denmark by around 1000–2000 per year, as compared to the main model, with prediction intervals being non-overlapping across the two models. The model based on log-linear trends (and Poisson regression) instead generally resulted in lower estimates of excess deaths for all countries than the main model. In particular for 2022, cardiovascular excess deaths in Finland, Norway, and Sweden decreased by about 1000 when using the log-linear model instead of the main model, with prediction intervals that were non-overlapping across this model and the main one. For estimates other than the aforementioned, prediction intervals were always overlapping when comparing estimates across the alternative models and the main one. However, substantial differences were still sometimes observed. Most notably, all-cause excess deaths differed by around 1000–2000 per year in Denmark and Sweden when comparing the model with five-year trends with the main model. Overall, the divergent results obtained from the different models underscore the importance of conducting sensitivity checks in studies of excess mortality, both with respect to functional form and the length of the reference period.

## Conclusion

In conclusion, the results of our investigation are broadly similar to evidence obtained from other contexts during the earlier phases of the COVID-19 pandemic, and point at a complex interplay between different factors that shaped excess mortality during the pandemic. While deaths due to COVID-19 constitute the most apparent consequence of the pandemic, our findings suggest the presence of several mechanisms, which may have caused the total annual excess death tolls to deviate from those pertaining to COVID-19—albeit to different extents in the different countries and over time. These mechanisms include substitution between different causes of death (deaths due to COVID-19 and deaths due to other respiratory diseases and dementia), substitution over time (COVID-19 deaths in 2020 leading to fewer dementia deaths in 2021 and 2022), and indirect consequences of COVID-19 infection and pandemic responses (such as cardiovascular death). Additional research may be able to shed more light on these mechanisms, and the extent to which they have been influenced by specific pandemic response policies. Additional research will also be needed to assess the continuing impact of the pandemic and how different population groups are affected in the longer term. Nevertheless, our study provides an informative account of how mortality may have been affected in the Nordic countries during the COVID-19 pandemic, with insights that could be instrumental when predicting or mitigating the consequences of future epidemics or pandemics.

## Supplementary Information

Below is the link to the electronic supplementary material.

Supplementary file1 (DOCX 10116 KB)

## Data Availability

All data used for the study are freely available online. Data on population sizes were obtained from Nordic Statistics (https://pxweb.nordicstatistics.org/pxweb/en/Nordic%20Statistics/Nordic%20Statistics__Demography__Population%20size/POPU01.px/). Data on causes of death were for Denmark obtained from Sundhedsdatastyrelsen (https://www.esundhed.dk/Emner/Hvad-doer-vi-af/Doedsaarsager#tabpanel97BF922C33EB4D969EA49D0D9D4ABC1A), for Finland from Tilastokeskus/Statistikcentralen (https://pxdata.stat.fi/PxWeb/pxweb/sv/StatFin/StatFin__ksyyt/statfin_ksyyt_pxt_11az.px/), for Norway from Statistikkbank (https://statistikkbank.fhi.no/dar/), and for Sweden from Socialstyrelsen (https://sdb.socialstyrelsen.se/if_dor/val.aspx). The data for the study were obtained on December 5, 2023.
