# Supplementary material for: Cause-specific excess mortality in Denmark, Finland, Norway, and Sweden during the COVID-19 pandemic 2020–2022: a study using nationwide population data
Source: Eur J Epidemiol. 2024 Sep 16;39(9):1037–50. doi: 10.1007/s10654-024-01154-0 (PMC11470911; doi:10.1007/s10654-024-01154-0)
Supplement: Supplementary file 1 — Supplementary file1 (DOCX 10116 KB) [file 10654_2024_1154_MOESM1_ESM.docx]

**Supplementary material to “Cause-specific excess mortality in Denmark, Finland, Norway, and Sweden during the COVID-19 pandemic 2020–2022: a study using nationwide population data”**

Anton Nilsson, Louise Emilsson, Kasper P. Kepp, Ann Kristin Skrindo Knudsen, Ingeborg Forthun, Christian Madsen, Jonas Björk, Tea Lallukka

2024-06-12

**Contents**

[Table S1: ICD codes 3](#_Toc170379500)

[Table S2: Excess mortality, with expected deaths obtained based on five-year linear trends 4](#_Toc170379501)

[Table S3: Excess mortality, with expected deaths obtained based on the log-linear model 6](#_Toc170379502)

[Table S4: Model performance 8](#_Toc170379503)

[Table S5: Excess mortality, with populations in 2021 and 2022 adjusted for previous excess deaths 9](#_Toc170379504)

[Table S6: Poisson regression vs linear regression with log-linear trends 11](#_Toc170379505)

[Fig. S1: Mortality trends among 0-19-year old males in Denmark 12](#_Toc170379506)

[Fig. S2: Mortality trends among 0-19-year old females in Denmark 13](#_Toc170379507)

[Fig. S3: Mortality trends among 20-44-year old males in Denmark 14](#_Toc170379508)

[Fig. S4: Mortality trends among 20-44-year old females in Denmark 15](#_Toc170379509)

[Fig. S5: Mortality trends among 45-69-year old males in Denmark 16](#_Toc170379510)

[Fig. S6: Mortality trends among 45-69-year old females in Denmark 17](#_Toc170379511)

[Fig. S7: Mortality trends among males aged 70 and older in Denmark 18](#_Toc170379512)

[Fig. S8: Mortality trends among females aged 70 and older in Denmark 19](#_Toc170379513)

[Fig. S9: Mortality trends among 0-19-year old males in Finland 20](#_Toc170379514)

[Fig. S10: Mortality trends among 0-19-year old females in Finland 21](#_Toc170379515)

[Fig. S11: Mortality trends among 20-44-year old males in Finland 22](#_Toc170379516)

[Fig. S12: Mortality trends among 20-44-year old females in Finland 23](#_Toc170379517)

[Fig. S13: Mortality trends among 45-69-year old males in Finland 24](#_Toc170379518)

[Fig. S14: Mortality trends among 45-69-year old females in Finland 25](#_Toc170379519)

[Fig. S15: Mortality trends among males aged 70 and older in Finland 26](#_Toc170379520)

[Fig. S16: Mortality trends among females aged 70 and older in Finland 27](#_Toc170379521)

[Fig. S17: Mortality trends among 0-39-year old males in Norway 28](#_Toc170379522)

[Fig. S18: Mortality trends among 0-39-year old females in Norway 29](#_Toc170379523)

[Fig. S19: Mortality trends among 40-69-year old males in Norway 30](#_Toc170379524)

[Fig. S20: Mortality trends among 40-69-year old females in Norway 31](#_Toc170379525)

[Fig. S21: Mortality trends among males aged 70 and older in Norway 32](#_Toc170379526)

[Fig. S22: Mortality trends among females aged 70 and older in Norway 33](#_Toc170379527)

[Fig. S23: Mortality trends among 0-19-year old males in Sweden 34](#_Toc170379528)

[Fig. S24: Mortality trends among 0-19-year old females in Sweden 35](#_Toc170379529)

[Fig. S25: Mortality trends among 20-44-year old males in Sweden 36](#_Toc170379530)

[Fig. S26: Mortality trends among 20-44-year old females in Sweden 37](#_Toc170379531)

[Fig. S27: Mortality trends among 45-69-year old males in Sweden 38](#_Toc170379532)

[Fig. S28: Mortality trends among 45-69-year old females in Sweden 39](#_Toc170379533)

[Fig. S29: Mortality trends among males aged 70 and older in Sweden 40](#_Toc170379534)

[Fig. S30: Mortality trends among females aged 70 and older in Sweden 41](#_Toc170379535)

[Fig. S31: Actual and expected deaths in Denmark 42](#_Toc170379536)

[Fig. S32: Actual and expected deaths in Finland 43](#_Toc170379537)

[Fig. S33: Actual and expected deaths in Norway 44](#_Toc170379538)

[Fig. S34: Actual and expected deaths in Sweden 45](#_Toc170379539)

# Table S1: ICD codes

|  | **Denmark** | **Finland** | **Norway** | **Sweden** |
| --- | --- | --- | --- | --- |
| COVID-19 | U07.1-2 | U07.1-2, U10.9 | U07.1-2 | U07.1-2, U10.9 |
| Respiratory (non-COVID-19) | J | J00-64, J66-99 | J | J |
| Cardiovascular | I | I00-I42.5, I42.8-I99 | I | I |
| Cancer | C00-D48 | C00-D48 | C00-D48 | C00-D48 |
| Dementia | F01, F03, G30, F00 | F01, F03, G30, R54 | F01, F03, G30 | F01, F03, G30 |
| Diabetes | E10-E14 | E10-E14 | E10-E14 | E10-E14 |
| External | V-Y | V01-X44, X46-Y89, U129 | V-Y | V-Y |

Notes: Total annual counts of U10.9 (“Multisystem inflammatory syndrome associated with COVID-19, unspecified”) were available for Norway as well. However, since the numbers were not reported for the age- and sex-strata, and as the counts were minimal (0 in 2020, 19 in 2021, and 5 in 2022), we chose to not include these in the analysis. For each country, diagnoses not included in the above categories were classified as “other.” The distribution of causes collected in the “other” category varied somewhat across the countries and over time, but diseases of the digestive system, diseases of the nervous system other than Alzheimer’s disease, and “symptoms, signs and abnormal clinical and laboratory findings, not elsewhere classified” typically ranked highest. For Denmark, “symptoms, signs and abnormal clinical and laboratory findings, not elsewhere classified” as well as “unknown” causes of deaths were particularly common, whereas for Finland, alcohol-related diseases was one of the most common causes of death among those collected in the “other” category.

# Table S2: Excess mortality, with expected deaths obtained based on five-year linear trends

| **Cause** | **Year** | **Denmark** | | | **Finland** | | | **Norway** | | | **Sweden** | | |
| --- | --- | --- | --- | --- | --- | --- | --- | --- | --- | --- | --- | --- | --- |
|  |  | **Deaths (actual & expected)** | **Absolute excess deaths (95% PI)** | **Relative excess deaths**  **(95% PI)** | **Deaths (actual & expected)** | **Absolute excess deaths (95% PI)** | **Relative excess deaths**  **(95% PI)** | **Deaths (actual & expected)** | **Absolute excess deaths (95% PI)** | **Relative excess deaths**  **(95% PI)** | **Deaths (actual & expected)** | **Absolute excess deaths (95% PI)** | **Relative excess deaths**  **(95% PI)** |
| All-cause | 2020 | 54,400;  54,966 | -566  (-1705; 573) | 0.99  (0.97; 1.01) | 55,498;  54,921 | 577  (-244; 1398) | 1.01  (1.00; 1.03) | 40,558;  40,711 | -153  (-774; 468) | 1.00  (0.98; 1.01) | 98,308;  90,184 | 8124  (6636; 9612) | 1.09  (1.07; 1.11) |
|  | 2021 | 56,870;  55,685 | 1185  (-169; 2539) | 1.02  (1.00; 1.05) | 57,632;  55,374 | 2258  (1290; 3226) | 1.04  (1.02; 1.06) | 41,713;  40,678 | 1035  (302; 1768) | 1.03  (1.01; 1.04) | 92,079;  89,781 | 2298  (554; 4042) | 1.03  (1.01; 1.05) |
|  | 2022 | 59,120;  56,434 | 2686  (1077; 4295) | 1.05  (1.02; 1.08) | 63,172;  55,406 | 7766  (6643; 8889) | 1.14  (1.12; 1.16) | 45,947;  40,491 | 5456  (4598; 6314) | 1.13  (1.11; 1.16) | 94,823;  89,742 | 5081  (3027; 7135) | 1.06  (1.03; 1.08) |
| COVID-19 | 2020 | 1075;  - | 1075  (-) | - | 558;  - | 558  (-) | - | 414;  - | 414  (-) | - | 9495;  - | 9495  (-) | - |
|  | 2021 | 1480;  - | 1480  (-) | - | 952;  - | 952  (-) | - | 864;  - | 864  (-) | - | 5321;  - | 5321  (-) | - |
|  | 2022 | 1590;  - | 1590  (-) | - | 4349;  - | 4349  (-) | - | 2858;  - | 2858  (-) | - | 3535;  - | 3535  (-) | - |
| Respiratory (non-COVID-19) | 2020 | 5720;  6600 | -880  (-1327; -433) | 0.87  (0.81; 0.93) | 1749;  2127 | -378  (-545; -211) | 0.82  (0.76; 0.89) | 3768;  4554 | -786  (-1021; -551) | 0.83  (0.78; 0.87) | 5550;  6611 | -1061  (-1510; -612) | 0.84  (0.78; 0.90) |
|  | 2021 | 5995;  6725 | -730  (-1261; -199) | 0.89  (0.82; 0.96) | 1766;  2155 | -389  (-587; -191) | 0.82  (0.74; 0.89) | 3660;  4599 | -939  (-1215; -663) | 0.80  (0.75; 0.84) | 5043;  6637 | -1594  (-2121; -1067) | 0.76  (0.7; 0.82) |
|  | 2022 | 6300;  6853 | -553  (-1184; 78) | 0.92  (0.83; 1.00) | 1940;  2174 | -234  (-465; -3) | 0.89  (0.80; 0.99) | 4401;  4625 | -224  (-543; 95) | 0.95  (0.89; 1.02) | 6173;  6692 | -519  (-1138; 100) | 0.92  (0.84; 1.01) |
| Cardio-vascular | 2020 | 11,560;  11,610 | -50  (-522; 422) | 1.00  (0.96; 1.04) | 18,496;  18,074 | 422  (1; 843) | 1.02  (1.00; 1.05) | 9577;  9136 | 441  (106; 776) | 1.05  (1.01; 1.09) | 28,111;  27,937 | 174  (-561; 909) | 1.01  (0.98; 1.03) |
|  | 2021 | 11,935;  11,326 | 609  (45; 1173) | 1.05  (1.00; 1.11) | 18,789;  17,690 | 1099  (595; 1603) | 1.06  (1.03; 1.09) | 10,205;  8618 | 1587  (1191; 1983) | 1.18  (1.13; 1.24) | 27,464;  26,927 | 537  (-325; 1399) | 1.02  (0.99; 1.05) |
|  | 2022 | 12,130;  11,023 | 1107  (435; 1779) | 1.10  (1.03; 1.17) | 19,526;  17,106 | 2420  (1830; 3010) | 1.14  (1.10; 1.18) | 10,381;  8035 | 2346  (1881; 2811) | 1.29  (1.22; 1.37) | 28,251;  26,013 | 2238  (1225; 3251) | 1.09  (1.04; 1.13) |
| Cancer | 2020 | 16,315;  16,372 | -57  (-384; 270) | 1.00  (0.98; 1.02) | 13,411;  13,384 | 27  (-369; 423) | 1.00  (0.97; 1.03) | 11,144;  11169 | -25  (-327; 277) | 1.00  (0.97; 1.02) | 23,532;  23,571 | -39  (-423; 345) | 1.00  (0.98; 1.01) |
|  | 2021 | 16,470;  16,436 | 34  (-354; 422) | 1.00  (0.98; 1.03) | 13,550;  13,562 | -12  (-475; 451) | 1.00  (0.97; 1.03) | 11,216;  11140 | 76  (-281; 433) | 1.01  (0.97; 1.04) | 23,070;  23,515 | -445  (-892; 2) | 0.98  (0.96; 1.00) |
|  | 2022 | 16,135;  16,490 | -355  (-812; 102) | 0.98  (0.95; 1.01) | 13,495;  13,688 | -193  (-730; 344) | 0.99  (0.95; 1.02) | 11,537;  11269 | 268  (-51; 587) | 1.02  (0.99; 1.05) | 23,436;  23,518 | -82  (-599; 435) | 1.00  (0.97; 1.02) |
| Dementia | 2020 | 4270;  4845 | -575  (-887; -263) | 0.88  (0.82; 0.94) | 10,673;  10,808 | -135  (-478; 208) | 0.99  (0.96; 1.02) | 4067;  4422 | -355  (-622; -88) | 0.92  (0.86; 0.98) | 9709;  10,053 | -344  (-760; 72) | 0.97  (0.93; 1.01) |
|  | 2021 | 4785;  5103 | -318  (-686; 50) | 0.94  (0.87; 1.01) | 11,455;  11,306 | 149  (-257; 555) | 1.01  (0.98; 1.05) | 4265;  4689 | -424  (-736; -112) | 0.91  (0.85; 0.97) | 8652;  10,399 | -1747  (-2239; -1255) | 0.83  (0.79; 0.87) |
|  | 2022 | 4805;  5379 | -574  (-1009; -139) | 0.89  (0.82; 0.97) | 12,313;  11,681 | 632  (158; 1106) | 1.05  (1.01; 1.10) | 4442;  4941 | -499  (-860; -138) | 0.90  (0.83; 0.96) | 9201;  10,830 | -1629  (-2211; -1047) | 0.85  (0.8; 0.9) |
| Diabetes | 2020 | 1275;  1363 | -88  (-215; 39) | 0.94  (0.85; 1.02) | 667;  617 | 50  (-26; 126) | 1.08  (0.95; 1.21) | 762;  566 | 196  (108; 284) | 1.35  (1.14; 1.56) | 2316;  2181 | 135  (-32; 302) | 1.06  (0.98; 1.14) |
|  | 2021 | 1355;  1355 | 0  (-153; 153) | 1.00  (0.89; 1.11) | 672;  644 | 28  (-62; 118) | 1.04  (0.90; 1.19) | 787;  555 | 232  (128; 336) | 1.42  (1.15; 1.68) | 2235;  2207 | 28  (-168; 224) | 1.01  (0.92; 1.10) |
|  | 2022 | 1340;  1347 | -7  (-189; 175) | 0.99  (0.86; 1.13) | 714;  669 | 45  (-61; 151) | 1.07  (0.90; 1.24) | 843;  542 | 301  (178; 424) | 1.56  (1.20; 1.91) | 2317;  2244 | 73  (-160; 306) | 1.03  (0.93; 1.14) |
| External | 2020 | 2188;  2321 | -133  (-247; -19) | 0.94  (0.9; 0.99) | 3084;  3302 | -218  (-438; 2) | 0.93  (0.87; 1.00) | 2638;  2701 | -63  (-220; 94) | 0.98  (0.92; 1.03) | 4871;  4990 | -119  (-340; 102) | 0.98  (0.93; 1.02) |
|  | 2021 | 2140;  2399 | -259  (-392; -126) | 0.89  (0.84; 0.94) | 3221;  3348 | -127  (-384; 130) | 0.96  (0.89; 1.04) | 2657;  2749 | -92  (-276; 92) | 0.97  (0.90; 1.03) | 4969;  4968 | 1  (-258; 260) | 1.00  (0.95; 1.05) |
|  | 2022 | 2348;  2485 | -137  (-294; 20) | 0.94  (0.89; 1.00) | 3147;  3382 | -235  (-535; 65) | 0.93  (0.85; 1.01) | 2747;  2794 | -47  (-263; 169) | 0.98  (0.91; 1.06) | 5196;  4964 | 232  (-72; 536) | 1.05  (0.98; 1.11) |
| Other | 2020 | 11,998;  11,854 | 143  (-323; 609) | 1.01  (0.97; 1.05) | 6860;  6608 | 252  (-3; 507) | 1.04  (1.00; 1.08) | 8188;  8136 | 52  (-201; 305) | 1.01  (0.98; 1.04) | 14,724;  14,842 | -118  (-461; 225) | 0.99  (0.97; 1.01) |
|  | 2021 | 12,710;  12,340 | 370  (-185; 925) | 1.03  (0.98; 1.08) | 7227;  6669 | 558  (258; 858) | 1.08  (1.03; 1.13) | 8059;  8302 | -243  (-543; 57) | 0.97  (0.94; 1.01) | 15,325;  15,128 | 197  (-205; 599) | 1.01  (0.99; 1.04) |
|  | 2022 | 14,473;  12,856 | 1617  (958; 2276) | 1.13  (1.07; 1.18) | 7688;  6705 | 983  (632; 1334) | 1.15  (1.09; 1.21) | 8738;  8442 | 296  (-57; 649) | 1.04  (0.99; 1.08) | 16,714;  15,481 | 1233  (765; 1701) | 1.08  (1.05; 1.11) |

Notes: Expected deaths were estimated based on strata-specific five-year trends (2015–2019). Prediction intervals (PIs) were obtained as estimates ± 1.96*standard error.

# Table S3: Excess mortality, with expected deaths obtained based on the log-linear model

| **Cause** | **Year** | **Denmark** | | | **Finland** | | | **Norway** | | | **Sweden** | | |
| --- | --- | --- | --- | --- | --- | --- | --- | --- | --- | --- | --- | --- | --- |
|  |  | **Deaths (actual & expected)** | **Absolute excess deaths (95% PI)** | **Relative excess deaths**  **(95% PI)** | **Deaths (actual & expected)** | **Absolute excess deaths (95% PI)** | **Relative excess deaths**  **(95% PI)** | **Deaths (actual & expected)** | **Absolute excess deaths (95% PI)** | **Relative excess deaths**  **(95% PI)** | **Deaths (actual & expected)** | **Absolute excess deaths (95% PI)** | **Relative excess deaths**  **(95% PI)** |
| All-cause | 2020 | 54,400;  53,911 | 489  (-603; 1581) | 1.01  (0.99; 1.03) | 55,498;  55,000 | 498  (-149; 1145) | 1.01  (1.00; 1.02) | 40,558;  40,824 | -266  (-916; 384) | 0.99  (0.98; 1.01) | 98,308;  91,429 | 6879  (5746; 8012) | 1.08  (1.06; 1.09) |
|  | 2021 | 56,870;  54,348 | 2522  (1360; 3684) | 1.05  (1.02; 1.07) | 57,632;  55,548 | 2084  (1398; 2770) | 1.04  (1.02; 1.05) | 41,713;  40,886 | 827  (144; 1510) | 1.02  (1.00; 1.04) | 92,079;  91,620 | 459  (-733; 1651) | 1.01  (0.99; 1.02) |
|  | 2022 | 59,120;  54,825 | 4295  (3050; 5540) | 1.08  (1.05; 1.10) | 63,172;  55,695 | 7477  (6754; 8200) | 1.13  (1.12; 1.15) | 45,947;  40,819 | 5128  (4412; 5844) | 1.13  (1.11; 1.15) | 94,823;  92,233 | 2590  (1324; 3856) | 1.03  (1.01; 1.04) |
| COVID-19 | 2020 | 1075;  - | 1075  (-) | - | 558;  - | 558  (-) | - | 414;  - | 414  (-) | - | 9495;  - | 9495  (-) | - |
|  | 2021 | 1480;  - | 1480  (-) | - | 952;  - | 952  (-) | - | 864;  - | 864  (-) | - | 5321;  - | 5321  (-) | - |
|  | 2022 | 1590;  - | 1590  (-) | - | 4349;  - | 4349  (-) | - | 2858;  - | 2858  (-) | - | 3535;  - | 3535  (-) | - |
| Respiratory (non-COVID-19) | 2020 | 5720;  6536 | -816  (-1147; -485) | 0.88  (0.83; 0.92) | 1749;  2084 | -335  (-460; -210) | 0.84  (0.79; 0.89) | 3768;  4640 | -872  (-1127; -617) | 0.81  (0.77; 0.86) | 5550;  6996 | -1446  (-1840; -1052) | 0.79  (0.75; 0.84) |
|  | 2021 | 5995;  6658 | -663  (-1020; -306) | 0.90  (0.85; 0.95) | 1766;  2111 | -345  (-478; -212) | 0.84  (0.78; 0.89) | 3660;  4737 | -1077  (-1350; -804) | 0.77  (0.73; 0.82) | 5043;  7187 | -2144  (-2569; -1719) | 0.70  (0.66; 0.74) |
|  | 2022 | 6300;  6785 | -485  (-871; -99) | 0.93  (0.88; 0.98) | 1940;  2130 | -190  (-333; -47) | 0.91  (0.85; 0.97) | 4401;  4820 | -419  (-710; -128) | 0.91  (0.86; 0.97) | 6173;  7418 | -1245  (-1708; -782) | 0.83  (0.78; 0.88) |
| Cardio-vascular | 2020 | 11,560;  11,799 | -239  (-596; 118) | 0.98  (0.95; 1.01) | 18,496;  18,342 | 154  (-165; 473) | 1.01  (0.99; 1.03) | 9577;  9565 | 12  (-279; 303) | 1.00  (0.97; 1.03) | 28,111;  28,662 | -551  (-1027; -75) | 0.98  (0.96; 1.00) |
|  | 2021 | 11,935;  11,672 | 263  (-107; 633) | 1.02  (0.99; 1.05) | 18,789;  18,157 | 632  (301; 963) | 1.03  (1.02; 1.05) | 10,205;  9274 | 931  (635; 1227) | 1.10  (1.07; 1.14) | 27,464;  28,050 | -586  (-1076; -96) | 0.98  (0.96; 1.00) |
|  | 2022 | 12,130;  11,553 | 577  (189; 965) | 1.05  (1.01; 1.09) | 19,526;  17,816 | 1710  (1367; 2053) | 1.10  (1.07; 1.12) | 10,381;  8951 | 1430  (1129; 1731) | 1.16  (1.12; 1.20) | 28,251;  27,600 | 651  (145; 1157) | 1.02  (1.00; 1.04) |
| Cancer | 2020 | 16,315;  16,437 | -122  (-412; 168) | 0.99  (0.98; 1.01) | 13,411;  13,344 | 67  (-237; 371) | 1.01  (0.98; 1.03) | 11,144;  11,333 | -189  (-442; 64) | 0.98  (0.96; 1.01) | 23,532;  24,143 | -611  (-1001; -221) | 0.97  (0.96; 0.99) |
|  | 2021 | 16,470;  16,544 | -74  (-382; 234) | 1.00  (0.98; 1.01) | 13,550;  13,520 | 30  (-293; 353) | 1.00  (0.98; 1.03) | 11,216;  11,382 | -166  (-431; 99) | 0.99  (0.96; 1.01) | 23,070;  24,321 | -1251  (-1665; -837) | 0.95  (0.93; 0.96) |
|  | 2022 | 16,135;  16,651 | -516  (-843; -189) | 0.97  (0.95; 0.99) | 13,495;  13,644 | -149  (-492; 194) | 0.99  (0.96; 1.01) | 11,537;  11,416 | 121  (-158; 400) | 1.01  (0.99; 1.04) | 23,436;  24,574 | -1138  (-1581; -695) | 0.95  (0.94; 0.97) |
| Dementia | 2020 | 4270;  4918 | -648  (-917; -379) | 0.87  (0.82; 0.92) | 10,673;  11,344 | -671  (-1112; -230) | 0.94  (0.90; 0.98) | 4067;  4472 | -405  (-635; -175) | 0.91  (0.86; 0.96) | 9709;  10,426 | -717  (-1091; -343) | 0.93  (0.90; 0.96) |
|  | 2021 | 4785;  5231 | -446  (-746; -146) | 0.91  (0.86; 0.97) | 11,455;  12,068 | -613  (-1103; -123) | 0.95  (0.91; 0.99) | 4265;  4787 | -522  (-780; -264) | 0.89  (0.84; 0.94) | 8652;  10,940 | -2288  (-2700; -1876) | 0.79  (0.76; 0.82) |
|  | 2022 | 4805;  5573 | -768  (-1103; -433) | 0.86  (0.81; 0.91) | 12,313;  12,683 | -370  (-911; 171) | 0.97  (0.93; 1.01) | 4442;  5099 | -657  (-946; -368) | 0.87  (0.82; 0.92) | 9201;  11,563 | -2362  (-2823; -1901) | 0.80  (0.76; 0.83) |
| Diabetes | 2020 | 1275;  1413 | -138  (-238; -38) | 0.90  (0.84; 0.97) | 667;  597 | 70  (-3; 143) | 1.12  (0.98; 1.25) | 762;  554 | 208  (150; 266) | 1.38  (1.23; 1.52) | 2316;  2185 | 131  (9; 253) | 1.06  (1.00; 1.12) |
|  | 2021 | 1355;  1434 | -79  (-187; 29) | 0.94  (0.87; 1.02) | 672;  623 | 49  (-33; 131) | 1.08  (0.94; 1.22) | 787;  544 | 243  (183; 303) | 1.45  (1.29; 1.61) | 2235;  2222 | 13  (-116; 142) | 1.01  (0.95; 1.06) |
|  | 2022 | 1340;  1456 | -116  (-232; 0) | 0.92  (0.85; 0.99) | 714;  647 | 67  (-25; 159) | 1.10  (0.95; 1.26) | 843;  532 | 311  (249; 373) | 1.58  (1.40; 1.77) | 2317;  2271 | 46  (-95; 187) | 1.02  (0.96; 1.08) |
| External | 2020 | 2188;  2158 | 30  (-92; 152) | 1.01  (0.96; 1.07) | 3084;  3094 | -10  (-163; 143) | 1.00  (0.95; 1.05) | 2638;  2618 | 20  (-109; 149) | 1.01  (0.96; 1.06) | 4871;  5190 | -319  (-507; -131) | 0.94  (0.9; 0.97) |
|  | 2021 | 2140;  2174 | -34  (-163; 95) | 0.98  (0.93; 1.04) | 3221;  3079 | 142  (-19; 303) | 1.05  (0.99; 1.10) | 2657;  2637 | 20  (-115; 155) | 1.01  (0.96; 1.06) | 4969;  5251 | -282  (-482; -82) | 0.95  (0.91; 0.98) |
|  | 2022 | 2348;  2196 | 151  (14; 288) | 1.07  (1.00; 1.14) | 3147;  3055 | 92  (-77; 261) | 1.03  (0.97; 1.09) | 2747;  2653 | 94  (-49; 237) | 1.04  (0.98; 1.09) | 5196;  5334 | -138  (-352; 76) | 0.97  (0.94; 1.01) |
| Other | 2020 | 11,998;  10,792 | 1206  (612; 1800) | 1.11  (1.05; 1.17) | 6860;  6538 | 322  (97; 547) | 1.05  (1.01; 1.09) | 8188;  7907 | 281  (20; 542) | 1.04  (1.00; 1.07) | 14,724;  14,308 | 416  (12; 820) | 1.03  (1.00; 1.06) |
|  | 2021 | 12,710;  10,907 | 1803  (1168; 2438) | 1.17  (1.1; 1.23) | 7227;  6571 | 656  (417; 895) | 1.10  (1.06; 1.14) | 8059;  7988 | 71  (-206; 348) | 1.01  (0.97; 1.04) | 15,325;  14,444 | 881  (452; 1310) | 1.06  (1.03; 1.09) |
|  | 2022 | 14,473;  11,037 | 3436  (2752; 4120) | 1.31  (1.23; 1.39) | 7688;  6581 | 1107  (852; 1362) | 1.17  (1.12; 1.21) | 8738;  8044 | 694  (401; 987) | 1.09  (1.05; 1.13) | 16,714;  14,645 | 2069  (1610; 2528) | 1.14  (1.11; 1.18) |

Notes: Expected deaths were estimated based on strata-specific log-linear ten-year trends (2010–2019). Prediction intervals (PIs) were obtained as estimates ± 1.96*standard error.

# Table S4: Model performance

| **Cause** | **Denmark** | | | **Finland** | | | **Norway** | | | **Sweden** | | |
| --- | --- | --- | --- | --- | --- | --- | --- | --- | --- | --- | --- | --- |
|  | **Main model** | **Five-year model** | **Log-linear model** | **Main model** | **Five-year model** | **Log-linear model** | **Main model** | **Five-year model** | **Log-linear model** | **Main model** | **Five-year model** | **Log-linear model** |
| All-cause | 0.469 | 0.257 | 0.467 | 0.508 | 0.252 | 0.502 | 0.709 | 0.290 | 0.707 | 0.368 | 0.340 | 0.367 |
| Respiratory (non-COVID-19) | 0.087 | 0.002 | 0.083 | 0.138 | -0.022 | 0.134 | 0.280 | 0.248 | 0.274 | 0.049 | -0.107 | 0.053 |
| Cardio-vascular | 0.331 | 0.212 | 0.338 | 0.368 | 0.219 | 0.366 | 0.438 | 0.375 | 0.435 | 0.343 | 0.221 | 0.341 |
| Cancer | 0.370 | 0.328 | 0.371 | 0.158 | -0.055 | 0.156 | 0.397 | 0.401 | 0.400 | 0.308 | 0.182 | 0.305 |
| Dementia | 0.090 | 0.077 | 0.090 | 0.095 | -0.024 | 0.093 | 0.618 | 0.642 | 0.612 | 0.061 | -0.010 | 0.061 |
| Diabetes | 0.115 | 0.014 | 0.112 | 0.018 | -0.046 | 0.008 | 0.298 | 0.192 | 0.298 | 0.001 | -0.024 | 0.005 |
| External | 0.069 | 0.087 | 0.069 | 0.273 | 0.038 | 0.265 | 0.156 | -0.024 | 0.153 | 0.135 | 0.168 | 0.142 |
| Other | 0.290 | 0.224 | 0.294 | 0.185 | -0.012 | 0.182 | 0.466 | 0.293 | 0.470 | 0.052 | 0.017 | 0.044 |

Notes: The table shows weighted averages of the adjusted R^2^ during the reference periods for different models, countries, and outcomes. Weights are proportional to the sizes of the involved age- and sex-strata in 2020. The main model (reported in Table 1 in the main text) uses a 10-year reference period (2010–2019) and assumes linear mortality trends. The five-year model (reported in Table S2) deviates from the main model by using a 5-year reference period (2015–2019). The log-linear model (reported in Table S3) instead deviates from the main model by assuming log-linear trends.

# Table S5: Excess mortality, with populations in 2021 and 2022 adjusted for previous excess deaths

| **Cause** | **Year** | **Denmark** | | | **Finland** | | | **Norway** | | | **Sweden** | | |
| --- | --- | --- | --- | --- | --- | --- | --- | --- | --- | --- | --- | --- | --- |
|  |  | **Deaths (actual & expected)** | **Absolute excess deaths (95% PI)** | **Relative excess deaths**  **(95% PI)** | **Deaths (actual & expected)** | **Absolute excess deaths (95% PI)** | **Relative excess deaths**  **(95% PI)** | **Deaths (actual & expected)** | **Absolute excess deaths (95% PI)** | **Relative excess deaths**  **(95% PI)** | **Deaths (actual & expected)** | **Absolute excess deaths (95% PI)** | **Relative excess deaths**  **(95% PI)** |
| All-cause | 2020 | 54,400; 53,567 | 833  (-372; 2038) | 1.02  (0.99; 1.04) | 55,498; 54,802 | 696  (-10; 1402) | 1.01  (1.00; 1.03) | 40,558; 40,590 | -32  (-751; 687) | 1.00  (0.98; 1.02) | 98,308; 91,196 | 7112  (5911; 8313) | 1.08  (1.06; 1.09) |
|  | 2021 | 56,870; 53,819 | 3051  (1752; 4350) | 1.06  (1.03; 1.08) | 57,632; 55,181 | 2451  (1692; 3210) | 1.04  (1.03; 1.06) | 41,713; 40,467 | 1246  (478; 2014) | 1.03  (1.01; 1.05) | 92,079; 92,042 | 37  (-1259; 1333) | 1.00  (0.99; 1.01) |
|  | 2022 | 59,120; 54,247 | 4873  (3462; 6284) | 1.09  (1.06; 1.12) | 63,172; 55,341 | 7831  (7020; 8642) | 1.14  (1.12; 1.16) | 45,947; 40,280 | 5667  (4846; 6488) | 1.14  (1.12; 1.16) | 94,823; 92,229 | 2594  (1204; 3984) | 1.03  (1.01; 1.04) |
|  | Sum | 170,390; 161,633 | 8757  (6491; 11,023) | 1.05  (1.04; 1.07) | 176,302; 165,324 | 10,978  (9663; 12,293) | 1.07  (1.06; 1.07) | 128,218; 121,336 | 6882  (5547; 8217) | 1.06  (1.05; 1.07) | 285,210; 275,467 | 9743  (7495; 11991) | 1.04  (1.03; 1.04) |
| COVID-19 | 2020 | 1075;  - | 1075  (-) | - | 558;  - | 558  (-) | - | 414;  - | 414  (-) | - | 9495  - | 9495  (-) | - |
|  | 2021 | 1480;  - | 1480  (-) | - | 952;  - | 952  (-) | - | 864;  - | 864  (-) | - | 5321  - | 5321  (-) | - |
|  | 2022 | 1590;  - | 1590  (-) | - | 4349;  - | 4349  (-) | - | 2858;  - | 2858  (-) | - | 3535  - | 3535  (-) | - |
|  | Sum | 4145;  - | 4145  (-) | - | 5859;  - | 5859  (-) | - | 4136;  - | 4136  (-) | - | 18,351  - | 18,351  (-) | - |
| Respira-tory (non-COVID-19) | 2020 | 5720;  6515 | -795  (-1136; -454) | 0.88  (0.83; 0.92) | 1749;  2067 | -318  (-459; -177) | 0.85  (0.79; 0.9) | 3768;  4639 | -871  (-1118; -624) | 0.81  (0.77; 0.86) | 5550;  6994 | -1444  (-1809; -1079) | 0.79  (0.75; 0.83) |
|  | 2021 | 5995;  6617 | -622  (-989; -255) | 0.91  (0.86; 0.96) | 1766;  2077 | -311  (-464; -158) | 0.85  (0.79; 0.91) | 3660;  4725 | -1065  (-1328; -802) | 0.77  (0.73; 0.82) | 5043;  7240 | -2197  (-2589; -1805) | 0.70  (0.66; 0.73) |
|  | 2022 | 6300;  6742 | -442  (-842; -42) | 0.93  (0.88; 0.99) | 1940;  2081 | -141  (-308; 26) | 0.93  (0.86; 1.01) | 4401;  4806 | -405  (-685; -125) | 0.92  (0.86; 0.97) | 6173;  7440 | -1267  (-1688; -846) | 0.83  (0.78; 0.88) |
|  | Sum | 18,015; 19,874 | -1859  (-2500; -1218) | 0.91  (0.88; 0.94) | 5455;  6225 | -770  (-1037; -503) | 0.88  (0.84; 0.91) | 11,829; 14,170 | -2341  (-2798; -1884) | 0.83  (0.81; 0.86) | 16,766; 21,674 | -4908  (-5590; -4226) | 0.77  (0.75; 0.8) |
| Cardio-vascular | 2020 | 11,560; 11,549 | 11  (-452; 474) | 1.00  (0.96; 1.04) | 18,496; 17,994 | 502  (116; 888) | 1.03  (1.01; 1.05) | 9577;  9235 | 342  (-3; 687) | 1.04  (1.00; 1.08) | 28,111; 28,128 | -17  (-566; 532) | 1.00  (0.98; 1.02) |
|  | 2021 | 11,935; 11,275 | 660  (160; 1160) | 1.06  (1.01; 1.11) | 18,789; 17,583 | 1206  (790; 1622) | 1.07  (1.04; 1.09) | 10,205; 8732 | 1473  (1105; 1841) | 1.17  (1.12; 1.22) | 27,464; 27,499 | -35  (-627; 557) | 1.00  (0.98; 1.02) |
|  | 2022 | 12,130; 11,022 | 1108  (565; 1651) | 1.10  (1.05; 1.15) | 19,526; 17,058 | 2468  (2019; 2917) | 1.14  (1.11; 1.17) | 10,381; 8188 | 2193  (1799; 2587) | 1.27  (1.21; 1.33) | 28,251; 26,552 | 1699  (1068; 2330) | 1.06  (1.04; 1.09) |
|  | Sum | 35,625; 33,846 | 1779  (909; 2649) | 1.05  (1.03; 1.08) | 56,811; 52,635 | 4176  (3453; 4899) | 1.08  (1.06; 1.09) | 30,163; 26,155 | 4008  (3367; 4649) | 1.15  (1.12; 1.18) | 83,826; 82,179 | 1647  (622; 2672) | 1.02  (1.01; 1.03) |
| Cancer | 2020 | 16,315; 16,352 | -37  (-358; 284) | 1.00  (0.98; 1.02) | 13,411; 13,338 | 73  (-248; 394) | 1.01  (0.98; 1.03) | 11,144; 11,275 | -131  (-409; 147) | 0.99  (0.96; 1.01) | 23,532; 24,092 | -560  (-974; -146) | 0.98  (0.96; 0.99) |
|  | 2021 | 16,470; 16,413 | 57  (-288; 402) | 1.00  (0.98; 1.02) | 13,550; 13,499 | 51  (-294; 396) | 1.00  (0.98; 1.03) | 11,216; 11,278 | -62  (-360; 236) | 0.99  (0.97; 1.02) | 23,070; 24,365 | -1295  (-1742; -848) | 0.95  (0.93; 0.96) |
|  | 2022 | 16,135; 16,500 | -365  (-739; 9) | 0.98  (0.96; 1.00) | 13,495; 13,634 | -139  (-507; 229) | 0.99  (0.96; 1.02) | 11,537; 11,274 | 263  (-56; 582) | 1.02  (0.99; 1.05) | 23,436; 24,552 | -1116  (-1598; -634) | 0.95  (0.94; 0.97) |
|  | Sum | 48,920; 49,266 | -345  (-947; 257) | 0.99  (0.98; 1.01) | 40,456; 40,471 | -15  (-613; 583) | 1.00  (0.98; 1.01) | 33,897; 33,828 | 69  (-448; 586) | 1.00  (0.99; 1.02) | 70,038; 73,009 | -2971  (-3747; -2195) | 0.96  (0.95; 0.97) |
| Dementia | 2020 | 4270;  4872 | -602  (-829; -375) | 0.88  (0.84; 0.92) | 10,673; 11,211 | -538  (-885; -191) | 0.95  (0.92; 0.98) | 4067;  4376 | -309  (-495; -123) | 0.93  (0.89; 0.97) | 9709; 10,303 | -594  (-900; -288) | 0.94  (0.91; 0.97) |
|  | 2021 | 4785;  5147 | -362  (-605; -119) | 0.93  (0.89; 0.97) | 11,455; 11,837 | -382  (-756; -8) | 0.97  (0.94; 1.00) | 4265;  4620 | -355  (-555; -155) | 0.92  (0.88; 0.96) | 8652; 10,879 | -2227  (-2558; -1896) | 0.80  (0.77; 0.82) |
|  | 2022 | 4805;  5467 | -662  (-927; -397) | 0.88  (0.84; 0.92) | 12,313; 12,409 | -96  (-496; 304) | 0.99  (0.96; 1.02) | 4442;  4865 | -423  (-635; -211) | 0.91  (0.87; 0.95) | 9201; 11,371 | -2170  (-2527; -1813) | 0.81  (0.78; 0.83) |
|  | Sum | 13,860; 15,486 | -1626  (-2051; -1201) | 0.90  (0.87; 0.92) | 34,441; 35,456 | -1016  (-1665; -367) | 0.97  (0.95; 0.99) | 12,774; 13,862 | -1088  (-1433; -743) | 0.92  (0.9; 0.94) | 27,562; 32,553 | -4991  (-5565; -4417) | 0.85  (0.83; 0.86) |
| Diabetes | 2020 | 1275;  1416 | -141  (-253; -29) | 0.90  (0.83; 0.97) | 667;  596 | 71  (10; 132) | 1.12  (1.01; 1.23) | 762;  545 | 217  (146; 288) | 1.40  (1.22; 1.58) | 2316;  2196 | 120  (-3; 243) | 1.05  (1.00; 1.11) |
|  | 2021 | 1355;  1430 | -75  (-198; 48) | 0.95  (0.87; 1.03) | 672;  614 | 58  (-9; 125) | 1.09  (0.98; 1.21) | 787;  526 | 261  (185; 337) | 1.05  (1.28; 1.71) | 2235;  2249 | -14  (-147; 119) | 0.99  (0.93; 1.05) |
|  | 2022 | 1340;  1449 | -109  (-244; 26) | 0.92  (0.84; 1.01) | 714;  630 | 84  (13; 155) | 1.13  (1.01; 1.26) | 843;  506 | 337  (255; 419) | 1.67  (1.39; 1.94) | 2317;  2292 | 25  (-118; 168) | 1.01  (0.95; 1.07) |
|  | Sum | 3970;  4295 | -325  (-539; -111) | 0.92  (0.88; 0.97) | 2053;  1839 | 213  (99; 327) | 1.12  (1.05; 1.19) | 2392;  1578 | 814  (681; 947) | 1.52  (1.39; 1.64) | 6868;  6736 | 132  (-99; 363) | 1.02  (0.98; 1.05) |
| External | 2020 | 2188;  2163 | 24  (-103; 151) | 1.01  (0.95; 1.07) | 3084;  3074 | 10  (-162; 182) | 1.00  (0.95; 1.06) | 2638;  2609 | 29  (-112; 170) | 1.01  (0.96; 1.07) | 4871;  5190 | -319  (-509; -129) | 0.94  (0.9; 0.97) |
|  | 2021 | 2140;  2175 | -35  (-172; 102) | 0.98  (0.92; 1.05) | 3221;  3038 | 183  (-1; 367) | 1.06  (1.00; 1.12) | 2657;  2619 | 38  (-113; 189) | 1.01  (0.96; 1.07) | 4969;  5273 | -304  (-508; -100) | 0.94  (0.91; 0.98) |
|  | 2022 | 2348;  2196 | 152  (5; 299) | 1.07  (1; 1.14) | 3147;  2998 | 149  (-47; 345) | 1.05  (0.98; 1.12) | 2747;  2629 | 118  (-41; 277) | 1.04  (0.98; 1.11) | 5196;  5336 | -140  (-358; 78) | 0.97  (0.93; 1.01) |
|  | Sum | 6676;  6534 | 141  (-96; 378) | 1.02  (0.98; 1.06) | 9452;  9110 | 342  (23; 661) | 1.04  (1.00; 1.07) | 8042;  7856 | 186  (-75; 447) | 1.02  (0.99; 1.06) | 15,036; 15,798 | -762  (-1115; -409) | 0.95  (0.93; 0.97) |
| Other | 2020 | 11,998; 10,700 | 1298  (667; 1929) | 1.12  (1.06; 1.19) | 6860;  6523 | 337  (94; 580) | 1.05  (1.01; 1.09) | 8188;  7884 | 304  (28; 580) | 1.04  (1.00; 1.07) | 14,724; 14,293 | 431  (12; 850) | 1.03  (1.00; 1.06) |
|  | 2021 | 12,710; 10,763 | 1947  (1269; 2625) | 1.18  (1.11; 1.26) | 7227;  6534 | 693  (432; 954) | 1.11  (1.06; 1.15) | 8059;  7938 | 121  (-173; 415) | 1.02  (0.98; 1.05) | 15,325; 14,538 | 787  (336; 1238) | 1.05  (1.02; 1.09) |
|  | 2022 | 14,473; 10,870 | 3602  (2865; 4339) | 1.33  (1.24; 1.42) | 7688;  6530 | 1158  (878; 1438) | 1.18  (1.13; 1.23) | 8738;  7984 | 754  (438; 1070) | 1.09  (1.05; 1.14) | 16,714; 14,687 | 2027  (1543; 2511) | 1.14  (1.10; 1.18) |
|  | Sum | 39,181; 32,333 | 6847  (5663; 8031) | 1.21  (1.17; 1.26) | 21,775; 19,587 | 2188  (1735; 2641) | 1.11  (1.09; 1.14) | 24,985; 23,807 | 1178  (666; 1690) | 1.05  (1.03; 1.07) | 46,763; 43,518 | 3245  (2461; 4029) | 1.07  (1.06; 1.09) |

Notes: Expected deaths were estimated based on strata-specific linear ten-year trends (2010–2019). Prediction intervals (PIs) were obtained as estimates ± 1.96*standard error.

# Table S6: Poisson regression versus linear regression with log-linear trends

| **Cause** | **Year** | **Denmark** | | **Finland** | | **Norway** | | **Sweden** | |
| --- | --- | --- | --- | --- | --- | --- | --- | --- | --- |
|  |  | **Poisson** | **Log-linear** | **Poisson** | **Log-linear** | **Poisson** | **Log-linear** | **Poisson** | **Log-linear** |
| All-cause | 2020 | 444 | 489 | 498 | 498 | -289 | -266 | 6847 | 6879 |
|  | 2021 | 2472 | 2522 | 2088 | 2084 | 801 | 827 | 426 | 459 |
|  | 2022 | 4239 | 4295 | 7485 | 7477 | 5100 | 5128 | 2557 | 2590 |
| Respiratory  (non-COVID-19) | 2020 | -835 | -816 | -358 | -335 | -880 | -872 | -1446 | -1446 |
|  | 2021 | -683 | -663 | -378 | -345 | -1086 | -1077 | -2139 | -2144 |
|  | 2022 | -508 | -485 | -251 | -190 | -428 | -419 | -1236 | -1245 |
| Cardiovascular | 2020 | -251 | -239 | 129 | 154 | -25 | 12 | -598 | -551 |
|  | 2021 | 252 | 263 | 604 | 632 | 890 | 931 | -638 | -586 |
|  | 2022 | 565 | 577 | 1680 | 1710 | 1385 | 1430 | 595 | 651 |
| Cancer | 2020 | -137 | -122 | 54 | 67 | -201 | -189 | -616 | -611 |
|  | 2021 | -89 | -74 | 17 | 30 | -178 | -166 | -1253 | -1251 |
|  | 2022 | -532 | -516 | -162 | -149 | 108 | 121 | -1139 | -1138 |
| Dementia | 2020 | -664 | -648 | -605 | -671 | -412 | -405 | -691 | -717 |
|  | 2021 | -466 | -446 | -526 | -613 | -530 | -522 | -2253 | -2288 |
|  | 2022 | -794 | -768 | -261 | -370 | -666 | -657 | -2318 | -2362 |
| Diabetes | 2020 | -151 | -138 | 63 | 70 | 202 | 208 | 113 | 131 |
|  | 2021 | -92 | -79 | 44 | 49 | 237 | 243 | -7 | 13 |
|  | 2022 | -131 | -116 | 63 | 67 | 305 | 311 | 23 | 46 |
| External | 2020 | 10 | 30 | -31 | -10 | 19 | 20 | -331 | -319 |
|  | 2021 | -55 | -34 | 120 | 142 | 20 | 20 | -293 | -282 |
|  | 2022 | 129 | 151 | 68 | 92 | 95 | 94 | -148 | -138 |
| Other | 2020 | 1155 | 1206 | 302 | 322 | 262 | 281 | 371 | 416 |
|  | 2021 | 1747 | 1803 | 636 | 656 | 49 | 71 | 831 | 881 |
|  | 2022 | 3373 | 3436 | 1089 | 1107 | 670 | 694 | 2014 | 2069 |

Notes: The table shows point predictions of absolute excess deaths based on Poisson regressions and on the log-linear models (the latter replicated from Table S3).


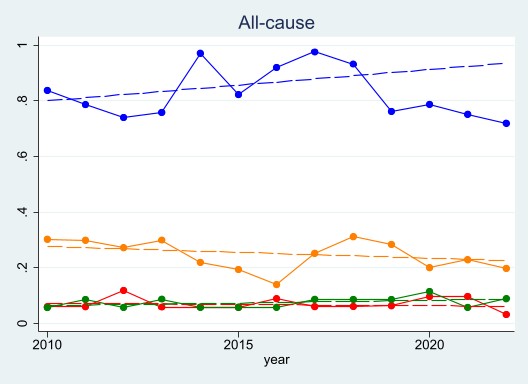

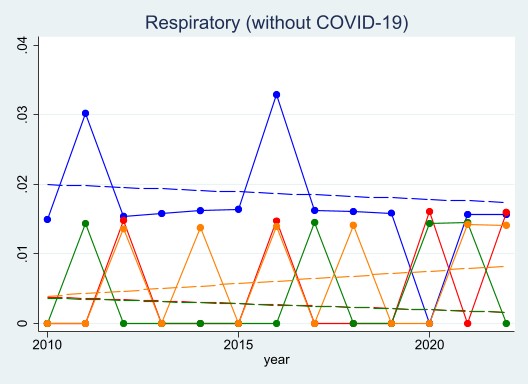

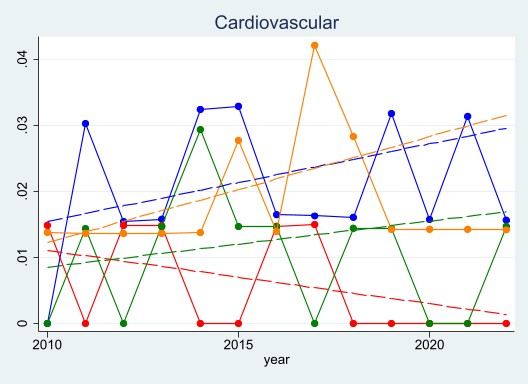

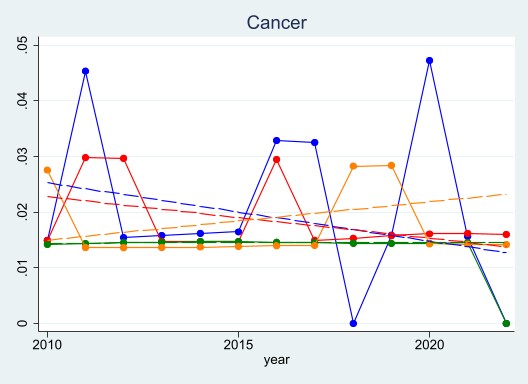

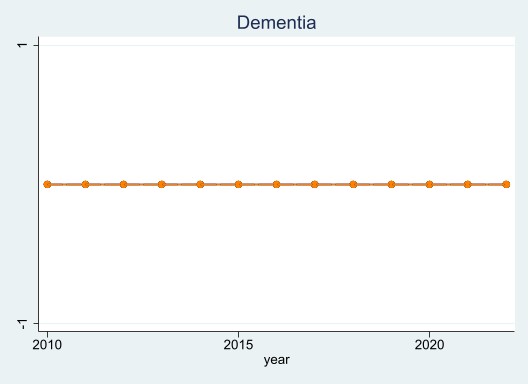

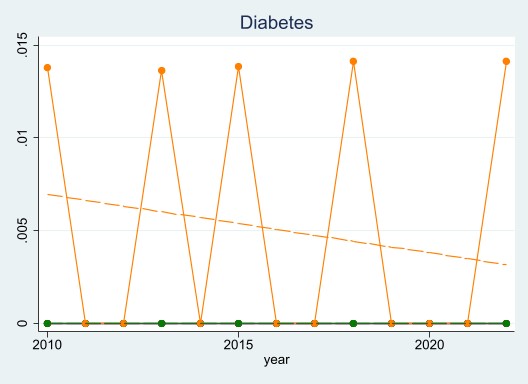

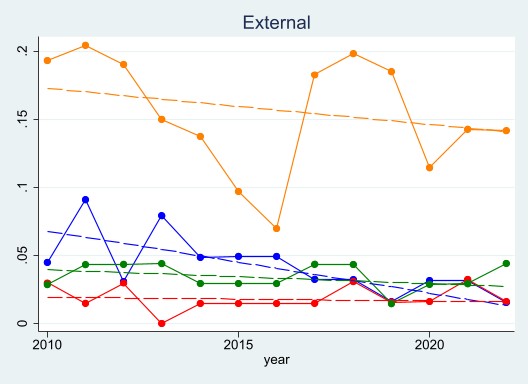

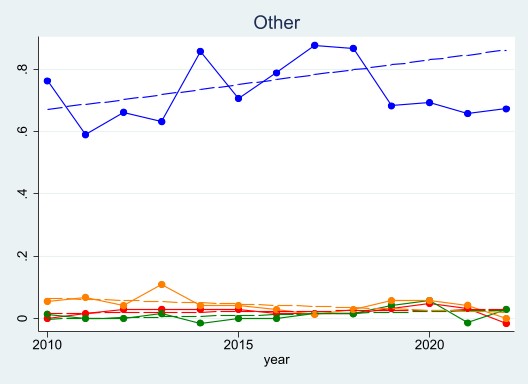


# Fig. S1: Mortality trends among 0-19-year old males in Denmark

Notes: The figure shows deaths per 1000 males in Denmark, separated by age group: 0-4-year-olds (blue), 5-9-year-olds (red), 10-14-year-olds (green), and 15-19-year-olds and older (orange). Linear trends were estimated based on 2010–2019.


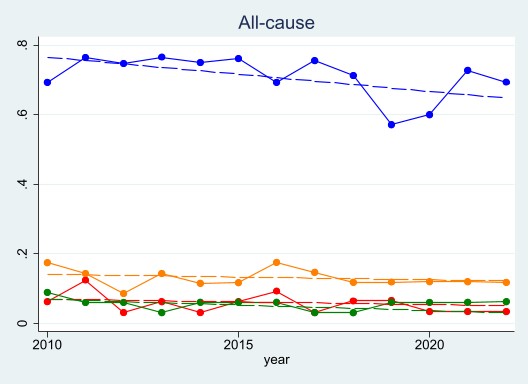

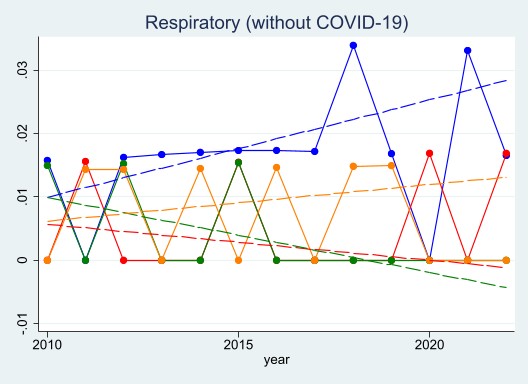

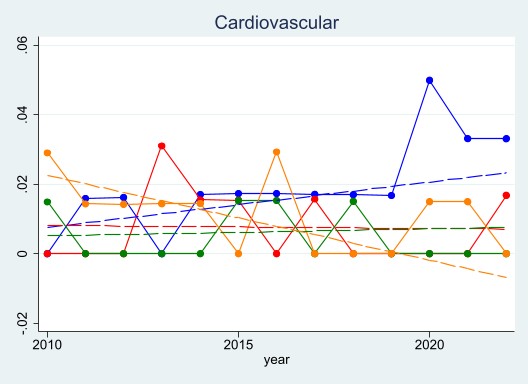

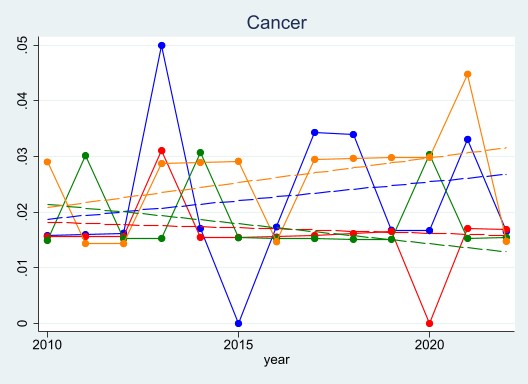

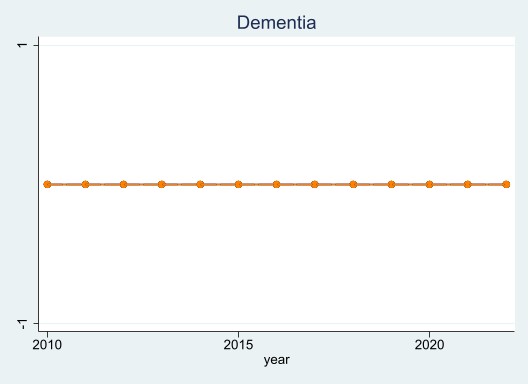

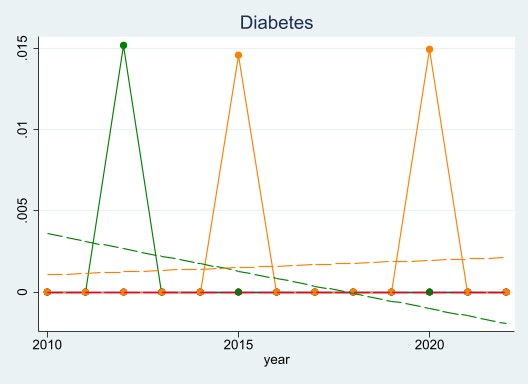

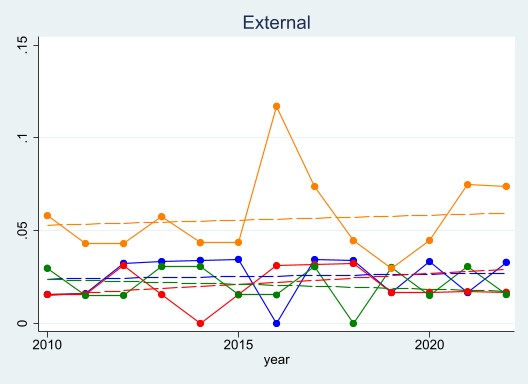

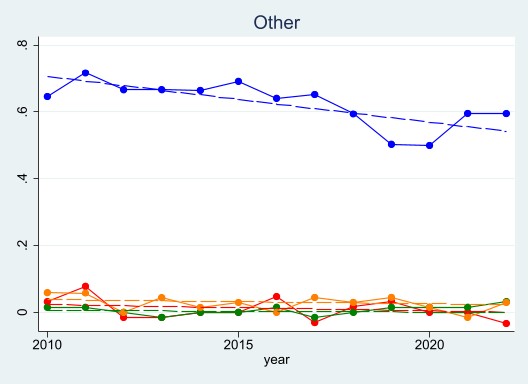


# Fig. S2: Mortality trends among 0-19-year old females in Denmark

Notes: The figure shows deaths per 1000 females in Denmark, separated by age group: 0-4-year-olds (blue), 5-9-year-olds (red), 10-14-year-olds (green), and 15-19-year-olds and older (orange). Linear trends were estimated based on 2010–2019.


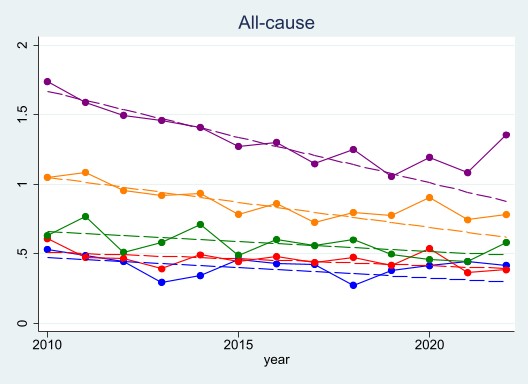

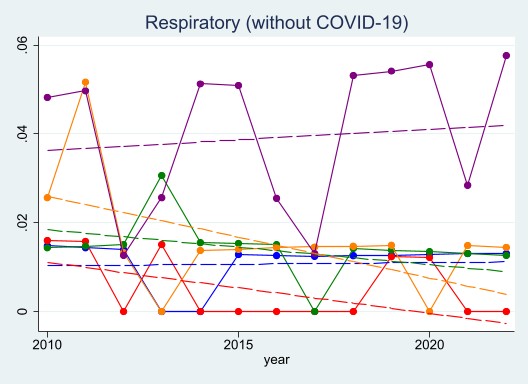

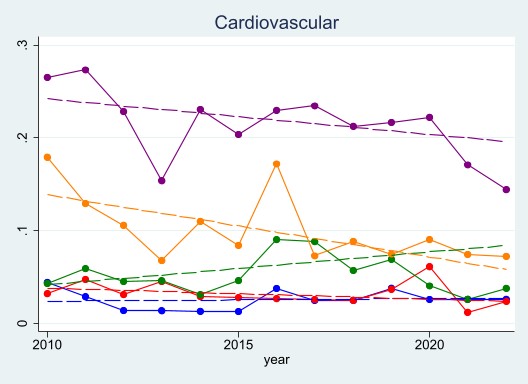

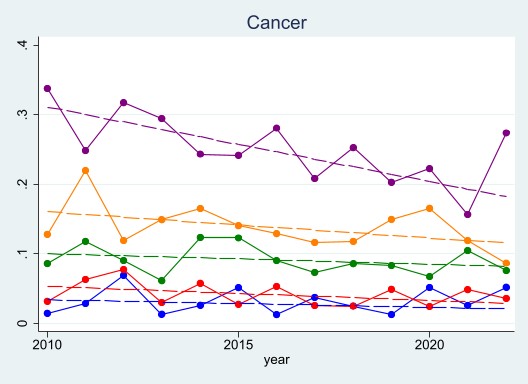

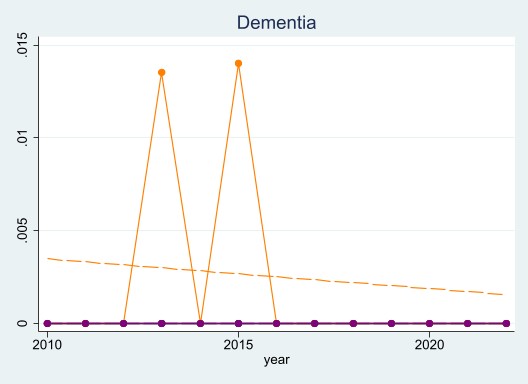

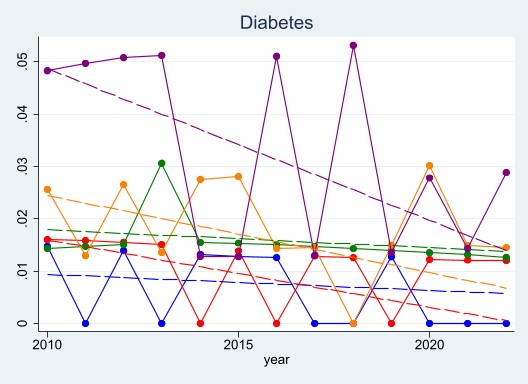

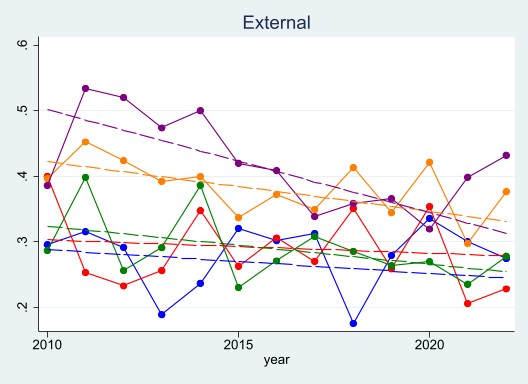

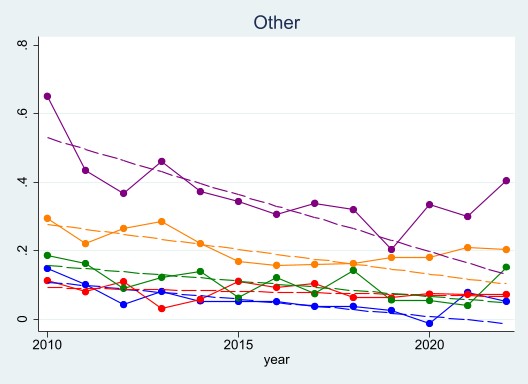


# Fig. S3: Mortality trends among 20-44-year old males in Denmark

Notes: The figure shows deaths per 1000 males in Denmark, separated by age group: 20-24-year-olds (blue), 25-29-year-olds (red), 30-34-year-olds (green), 35-39-year-olds (orange), and 40-44-year-olds and older (purple). Linear trends were estimated based on 2010–2019.


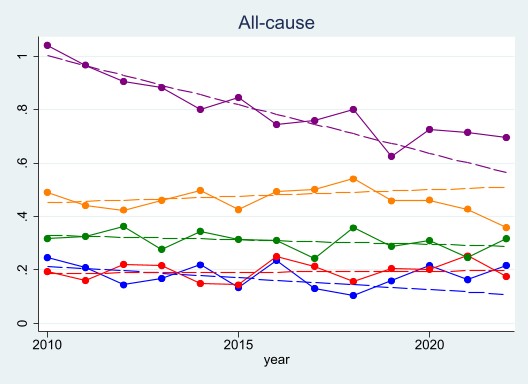

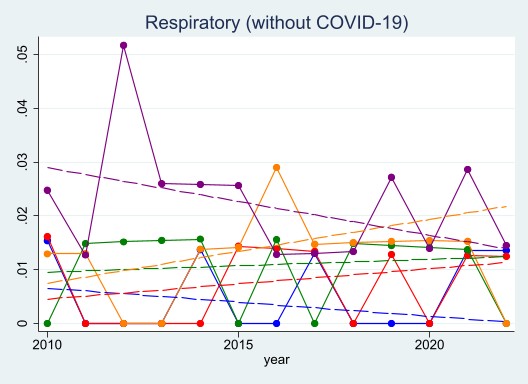

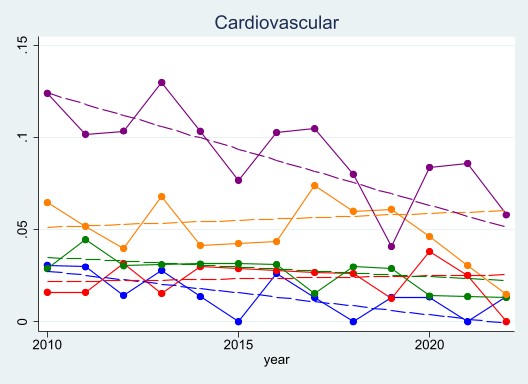

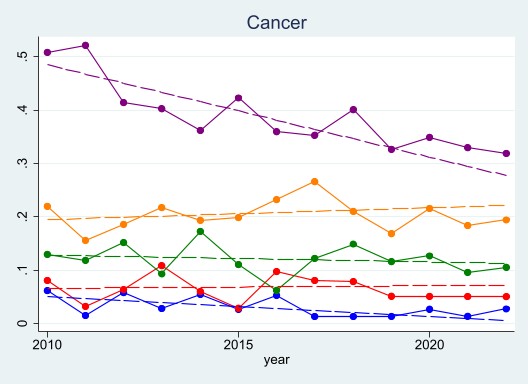

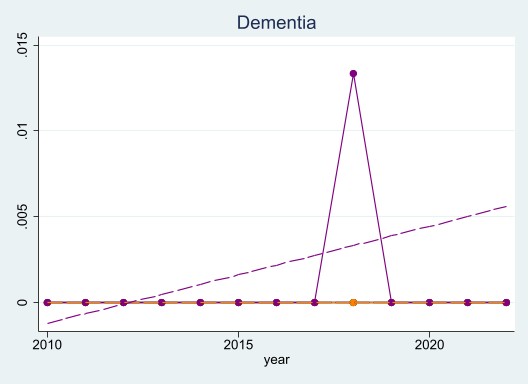

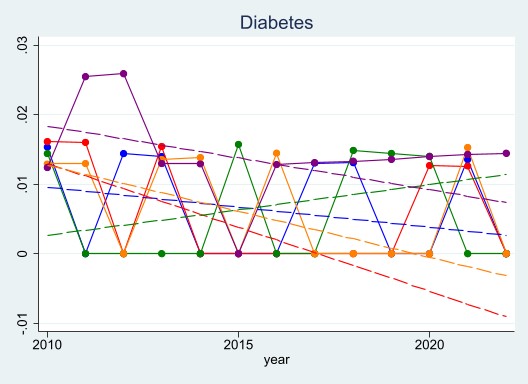

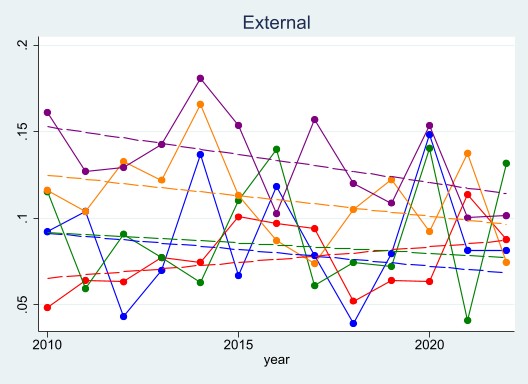

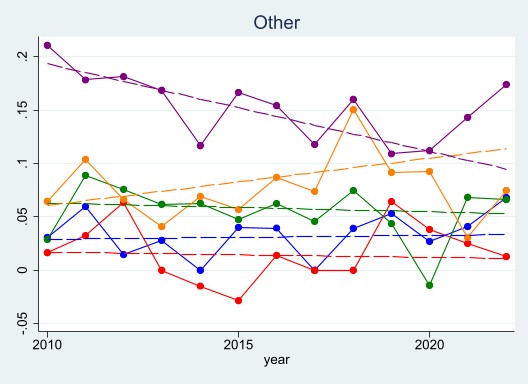


# Fig. S4: Mortality trends among 20-44-year old females in Denmark

Notes: The figure shows deaths per 1000 males in Denmark, separated by age group: 20-24-year-olds (blue), 25-29-year-olds (red), 30-34-year-olds (green), 35-39-year-olds (orange), and 40-44-year-olds and older (purple). Linear trends were estimated based on 2010–2019.


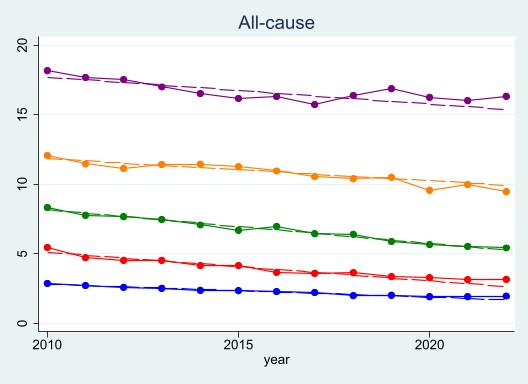

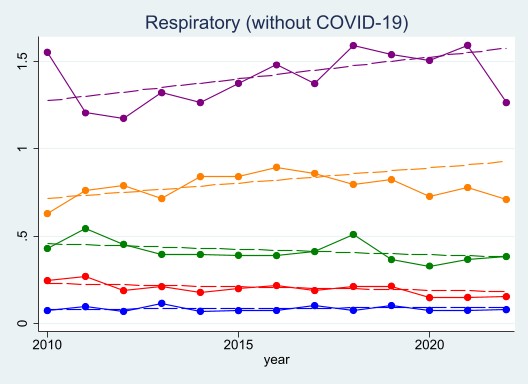

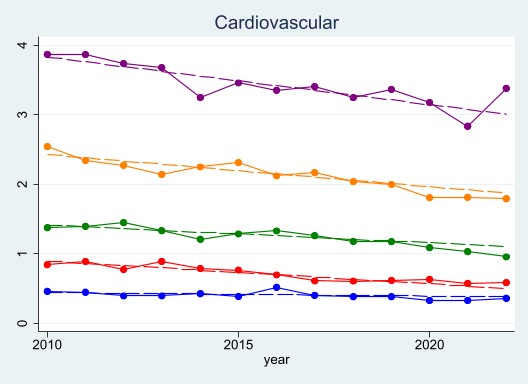

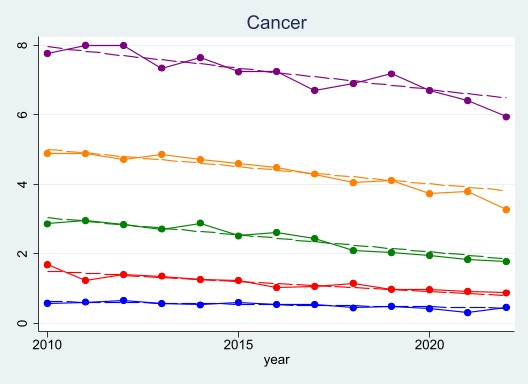

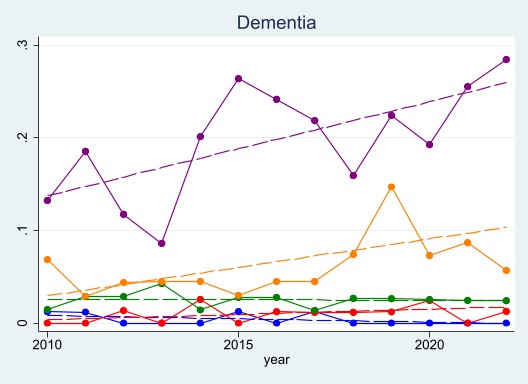

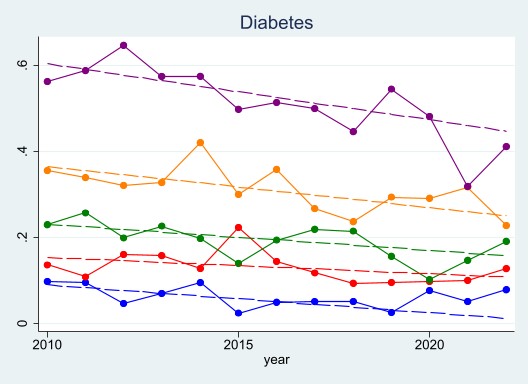

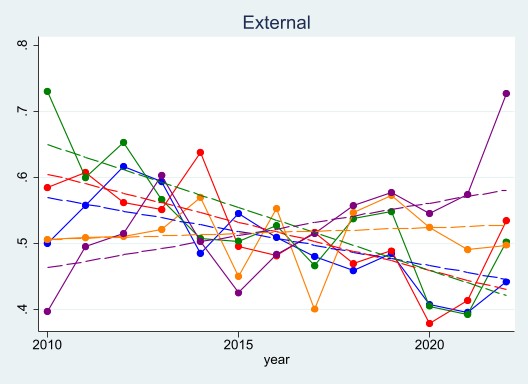

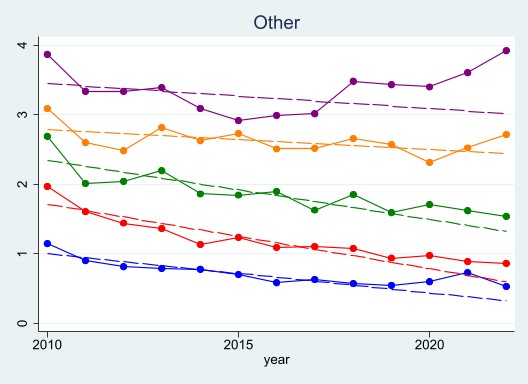


# Fig. S5: Mortality trends among 45-69-year old males in Denmark

Notes: The figure shows deaths per 1000 males in Denmark, separated by age group: 45-49-year-olds (blue), 50-54-year-olds (red), 55-59-year-olds (green), 60-64-year-olds (orange), and 65-69-year-olds and older (purple). Linear trends were estimated based on 2010–2019.


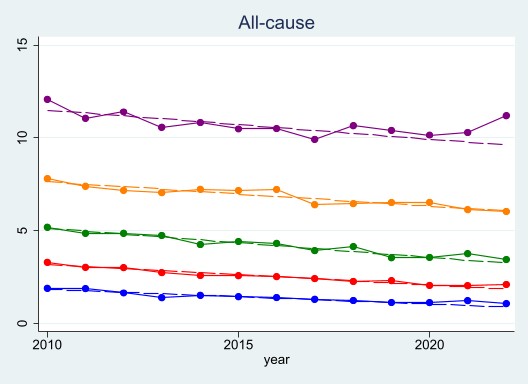

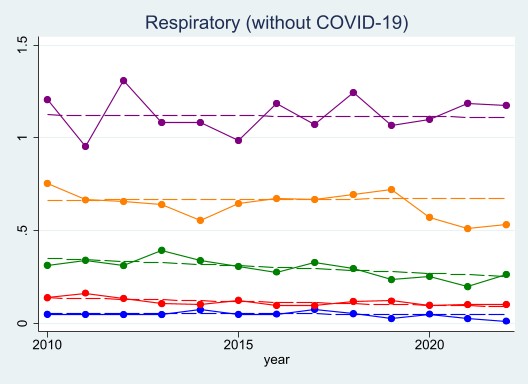

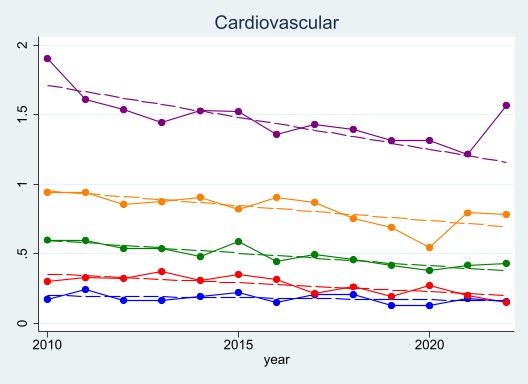

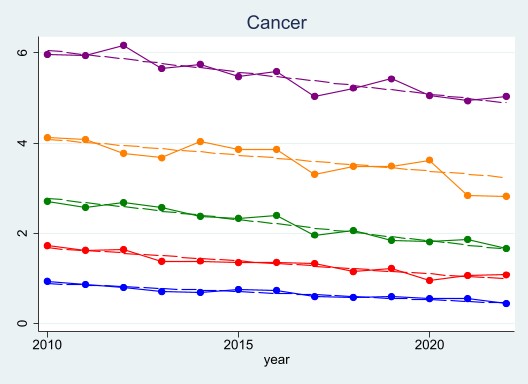

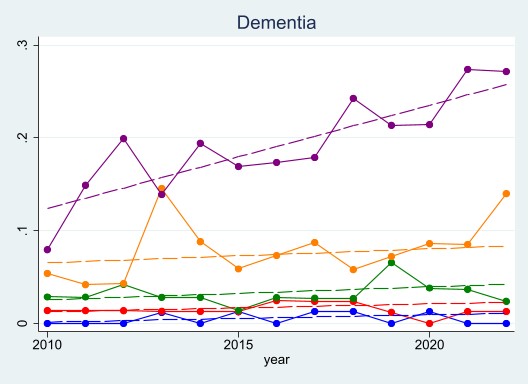

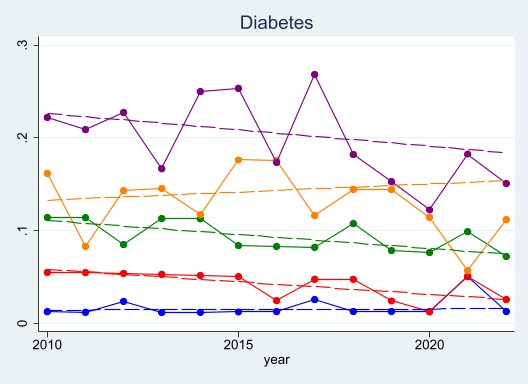

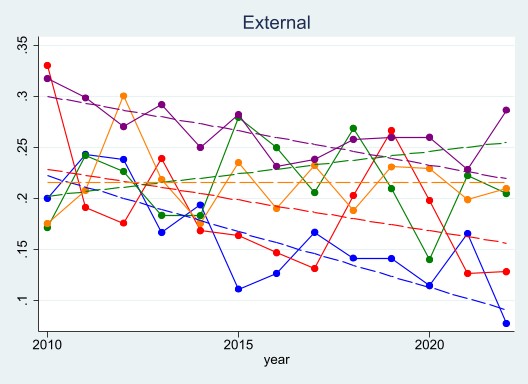

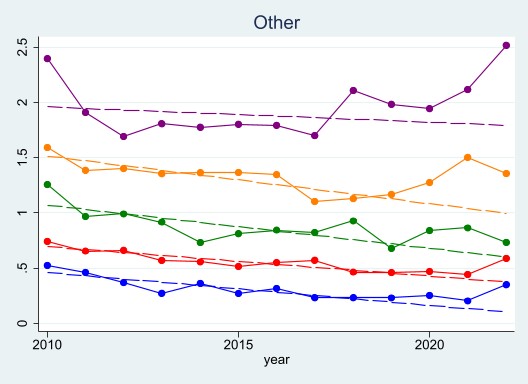


# Fig. S6: Mortality trends among 45-69-year old females in Denmark

Notes: The figure shows deaths per 1000 females in Denmark, separated by age group: 45-49-year-olds (blue), 50-54-year-olds (red), 55-59-year-olds (green), 60-64-year-olds (orange), and 65-69-year-olds and older (purple). Linear trends were estimated based on 2010–2019.


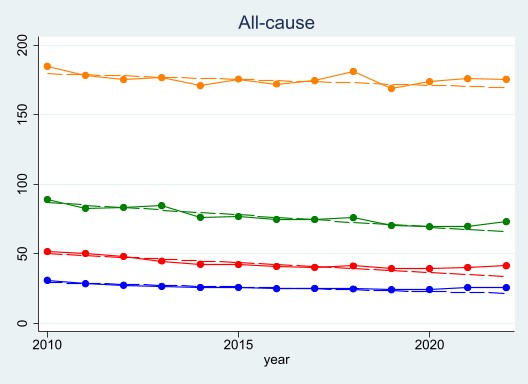

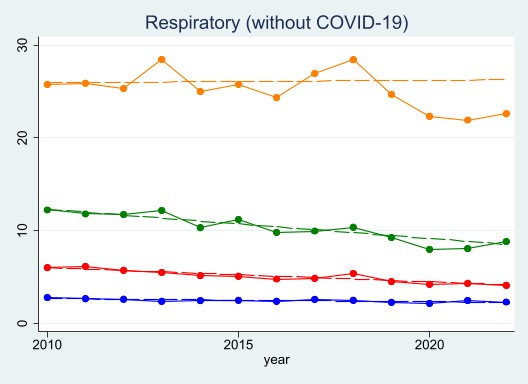

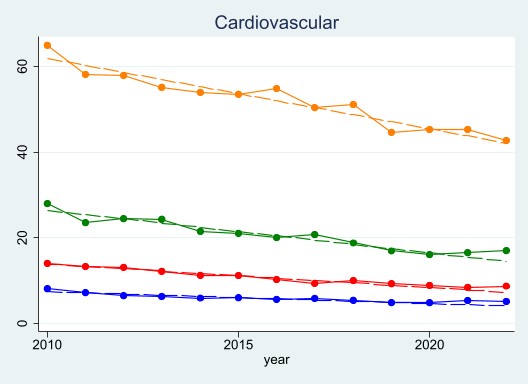

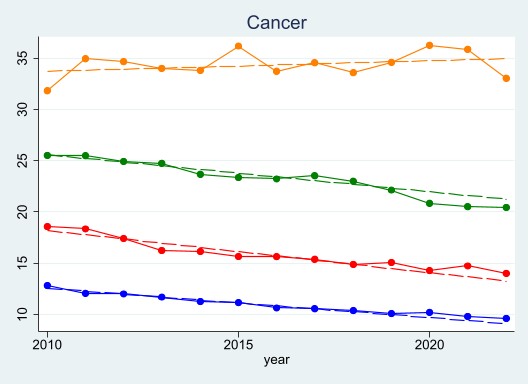

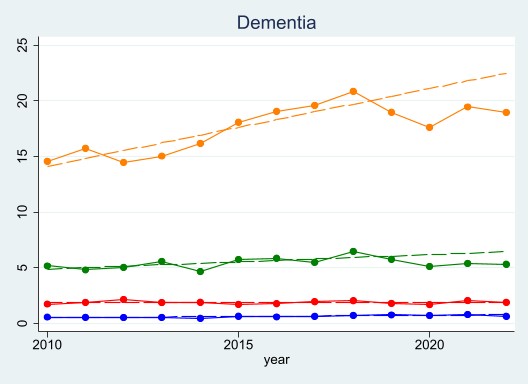

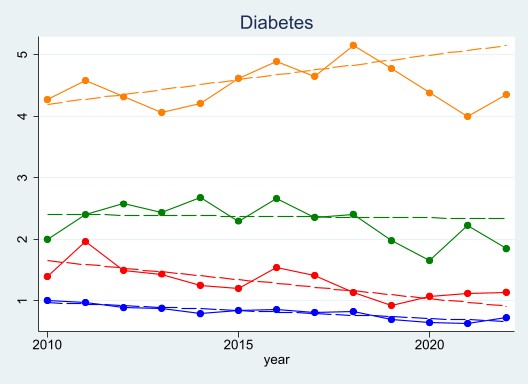

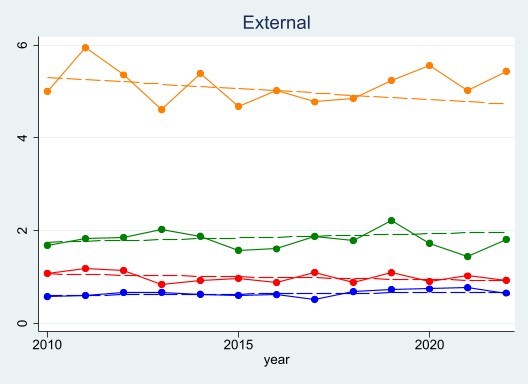

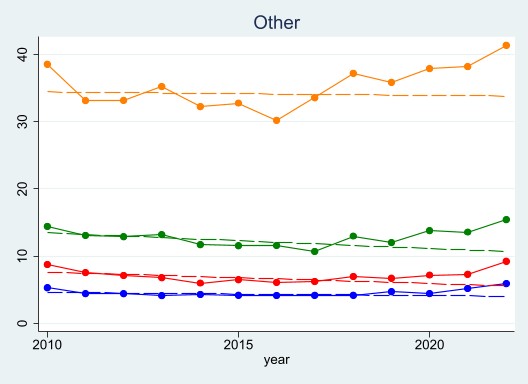


# Fig. S7: Mortality trends among males aged 70 and older in Denmark

Notes: The figure shows deaths per 1000 males in Denmark, separated by age group: 70-74-year-olds (blue), 75-79-year-olds (red), 80-84-year-olds (green), and 85-year-olds and older (orange). Linear trends were estimated based on 2010–2019.


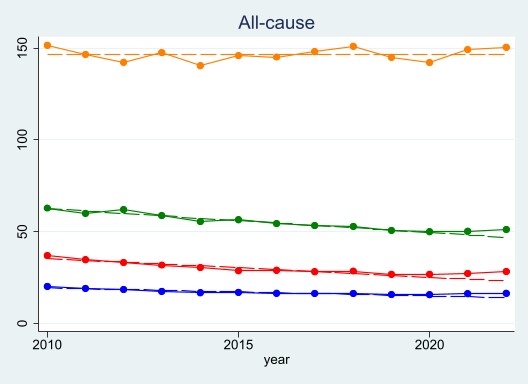

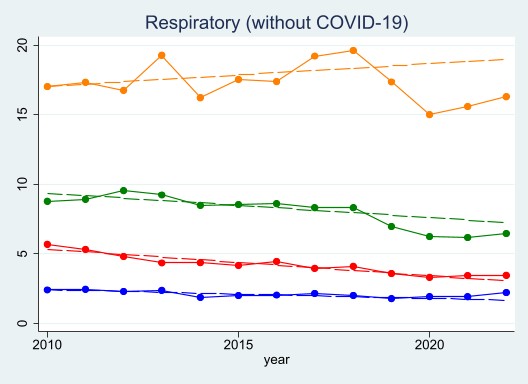

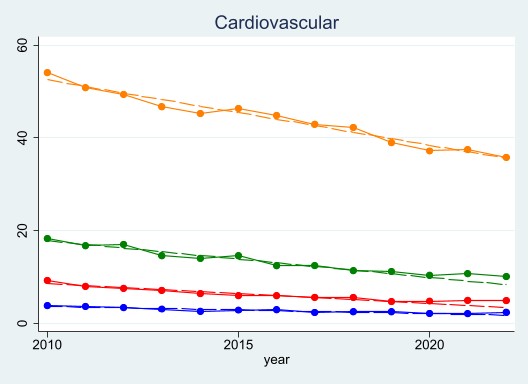

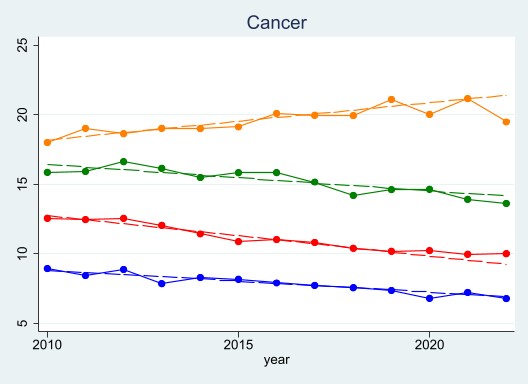

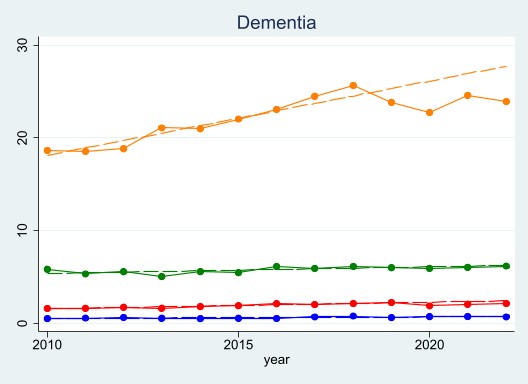

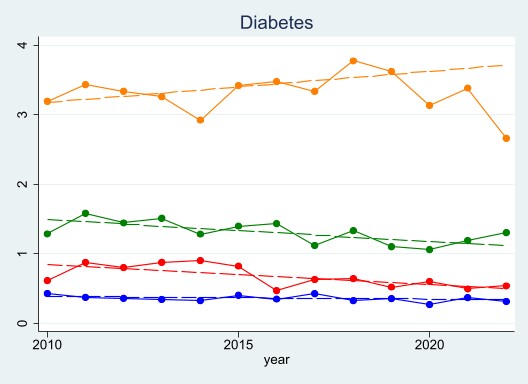

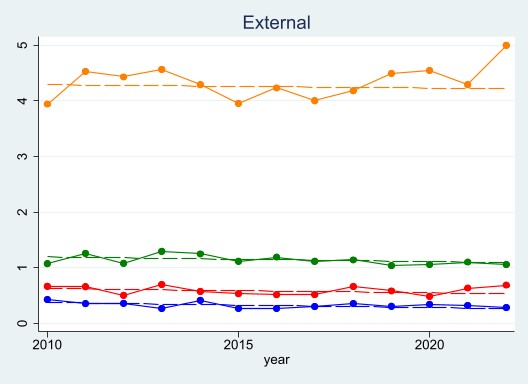

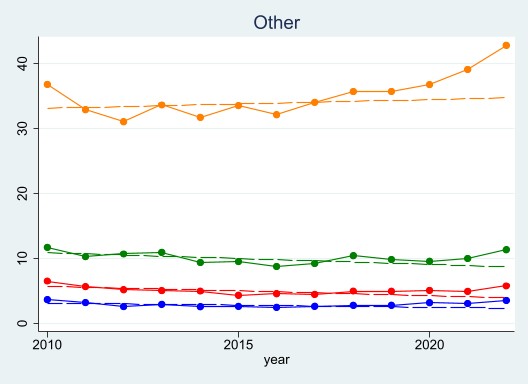


# Fig. S8: Mortality trends among females aged 70 and older in Denmark

Notes: The figure shows deaths per 1000 females in Denmark, separated by age group: 70-74-year-olds (blue), 75-79-year-olds (red), 80-84-year-olds (green), and 85-year-olds and older (orange). Linear trends were estimated based on 2010–2019.


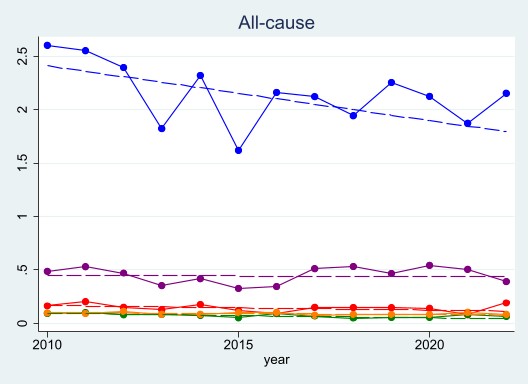

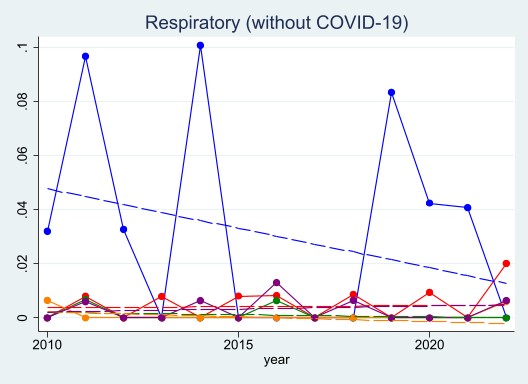

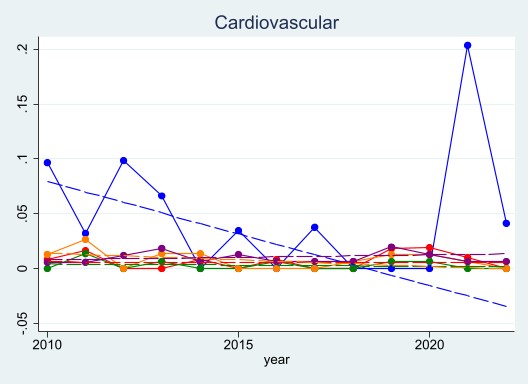

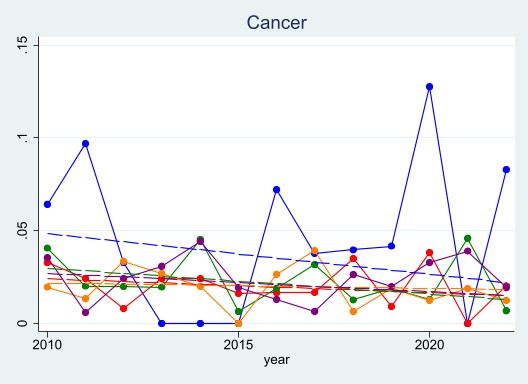

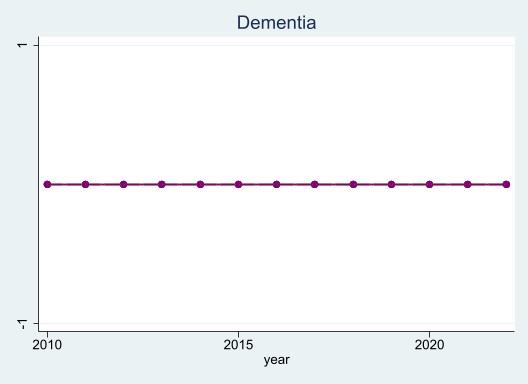

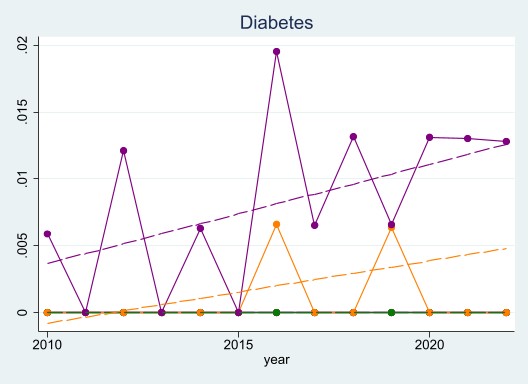

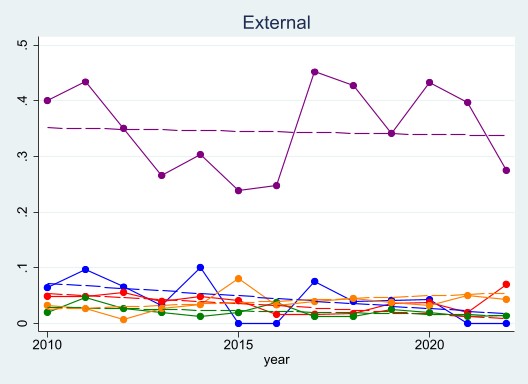

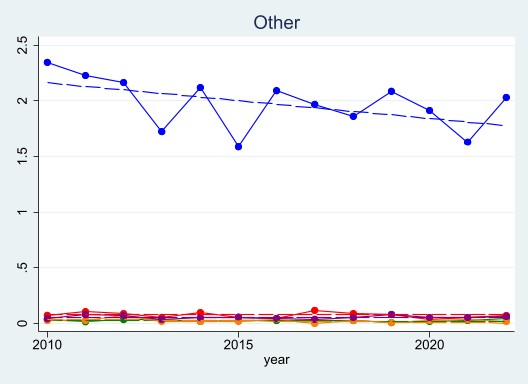


# Fig. S9: Mortality trends among 0-19-year old males in Finland

Notes: The figure shows deaths per 1000 males in Finland, separated by age group: 0-year-olds (blue), 1-4-year-olds (red), 5-9-year-olds (green), 10-14-year-olds (orange), and 15-19-year-olds and older (purple). Linear trends were estimated based on 2010–2019.


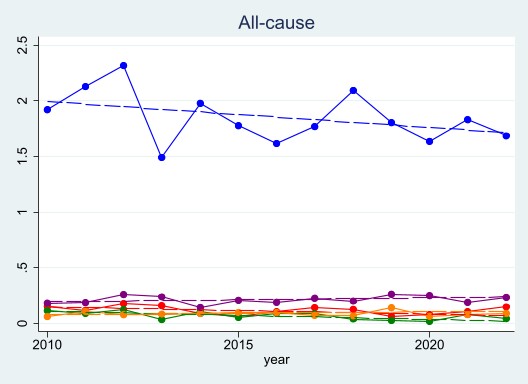

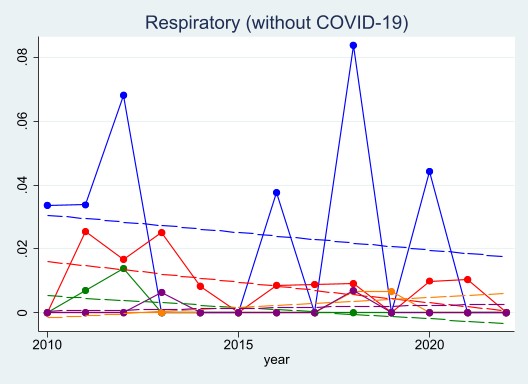

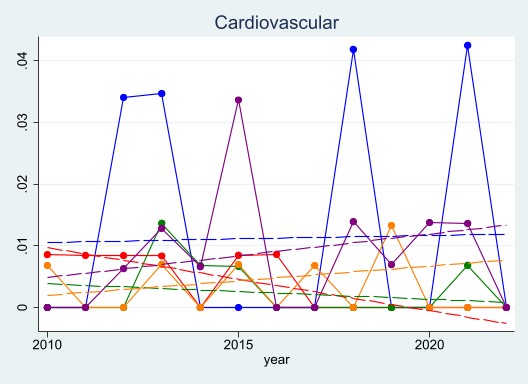

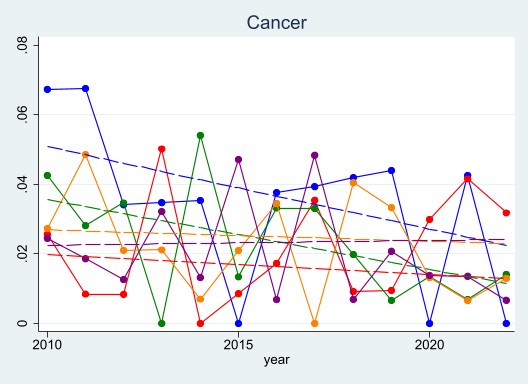

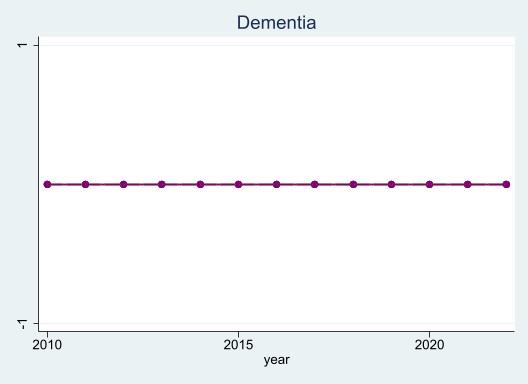

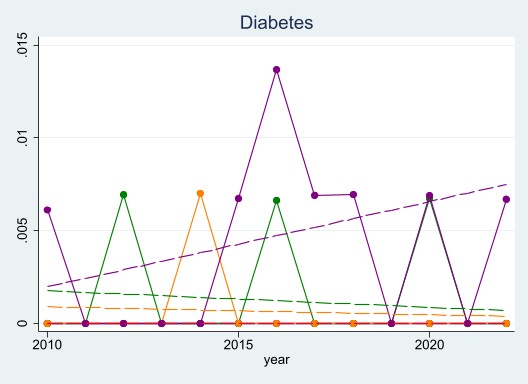

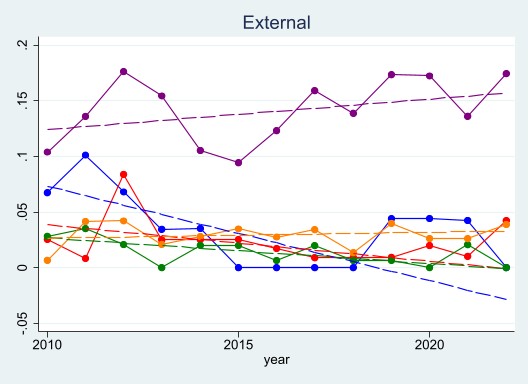

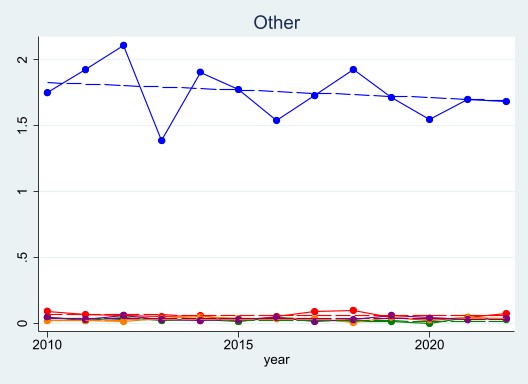


# Fig. S10: Mortality trends among 0-19-year old females in Finland

Notes: The figure shows deaths per 1000 females in Finland, separated by age group: 0-year-olds (blue), 1-4-year-olds (red), 5-9-year-olds (green), 10-14-year-olds (orange), and 15-19-year-olds and older (purple). Linear trends were estimated based on 2010–2019.


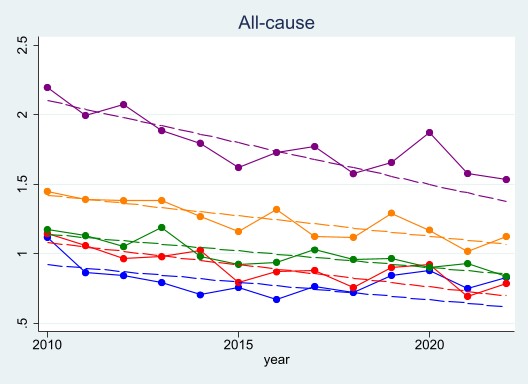

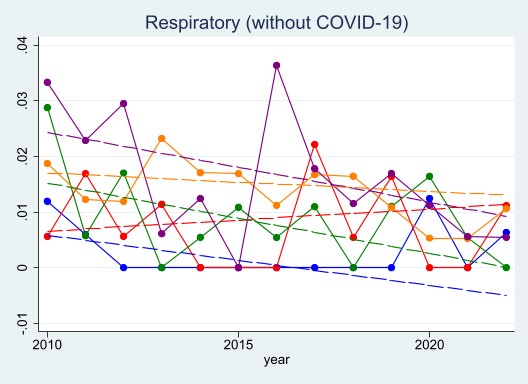

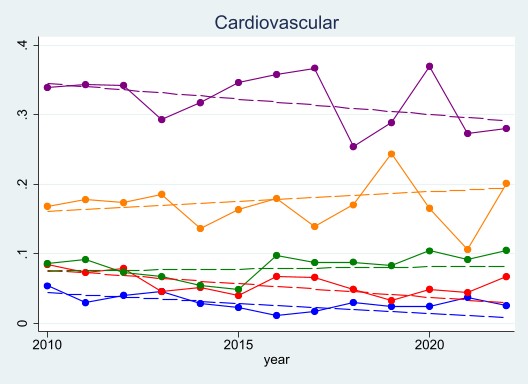

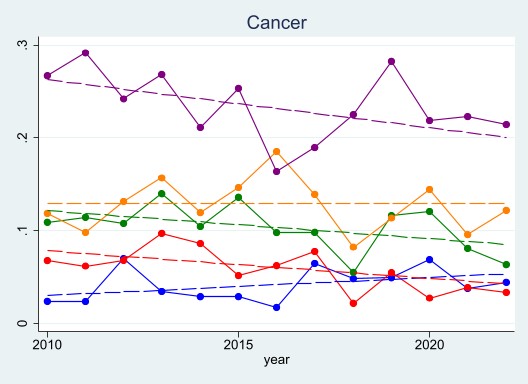

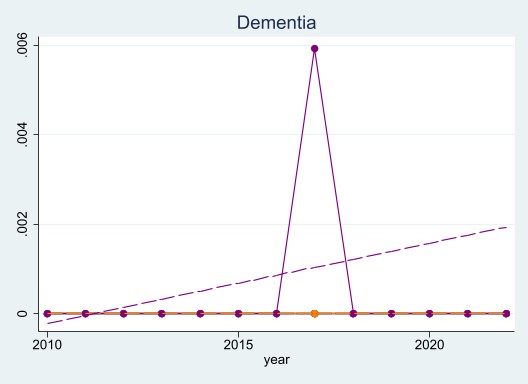

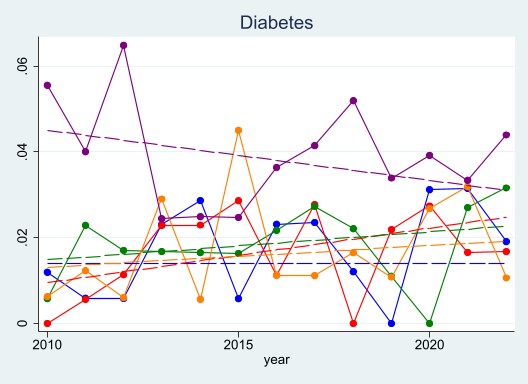

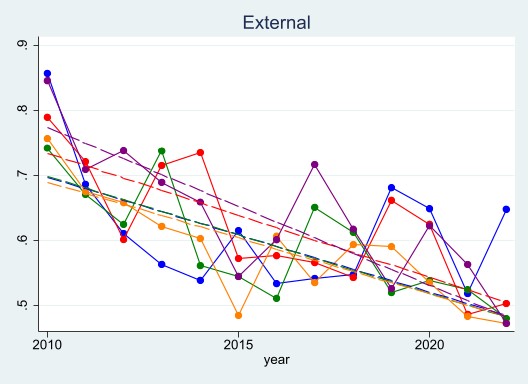

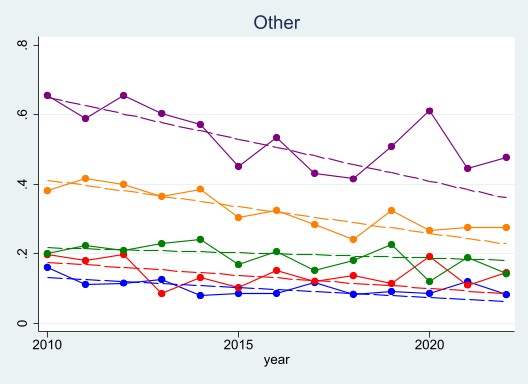


# Fig. S11: Mortality trends among 20-44-year old males in Finland

Notes: The figure shows deaths per 1000 males in Finland, separated by age group: 20-24-year-olds (blue), 25-29-year-olds (red), 30-34-year-olds (green), 35-39-year-olds (orange), and 40-44-year-olds and older (purple). Linear trends were estimated based on 2010–2019.


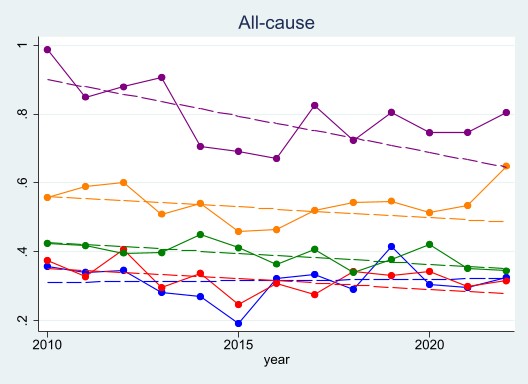

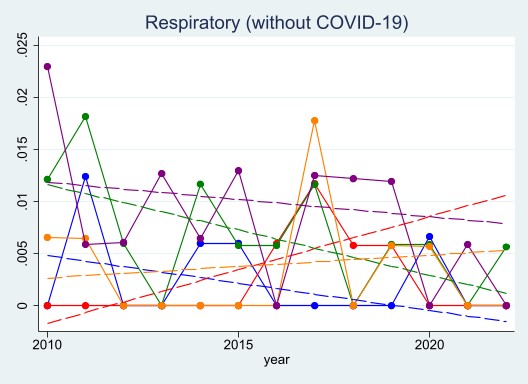

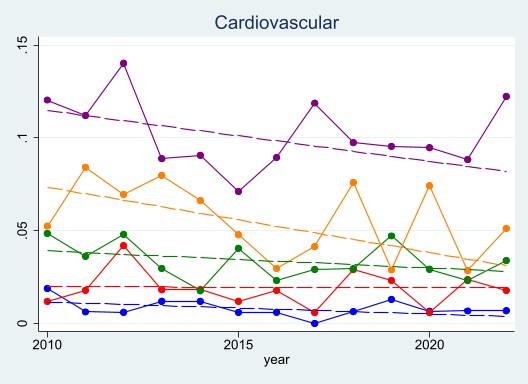

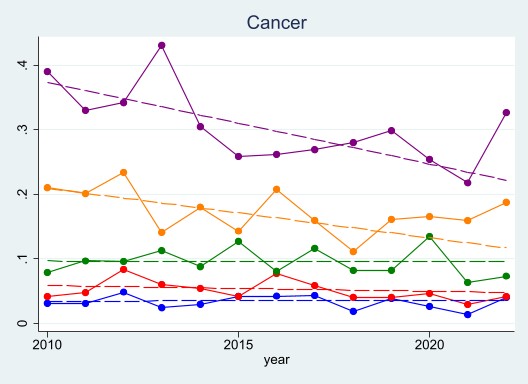

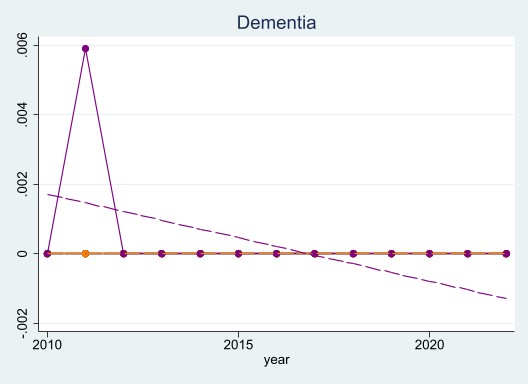

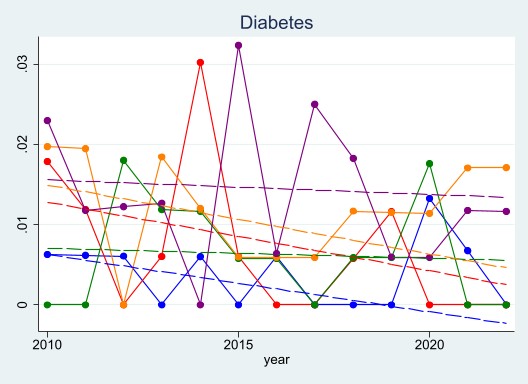

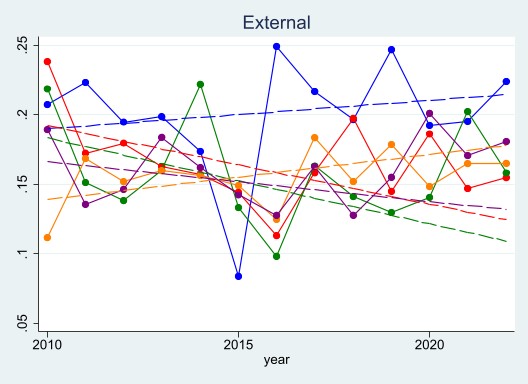

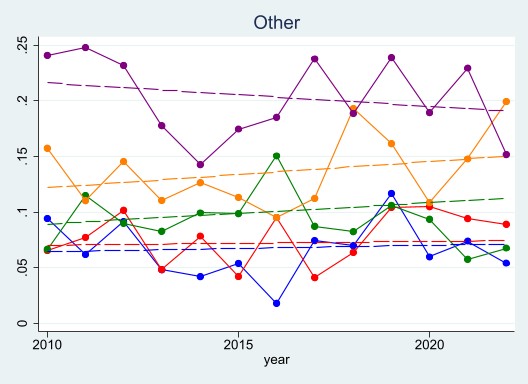


# Fig. S12: Mortality trends among 20-44-year old females in Finland

Notes: The figure shows deaths per 1000 females in Finland, separated by age group: 20-24-year-olds (blue), 25-29-year-olds (red), 30-34-year-olds (green), 35-39-year-olds (orange), and 40-44-year-olds and older (purple). Linear trends were estimated based on 2010–2019.


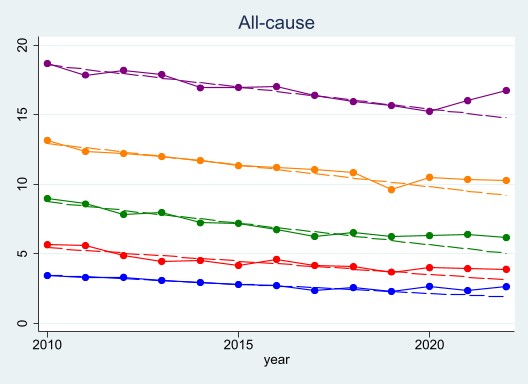

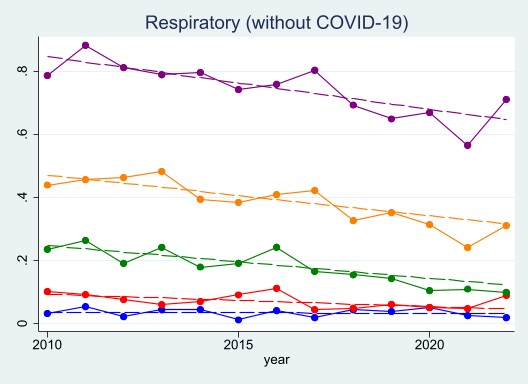

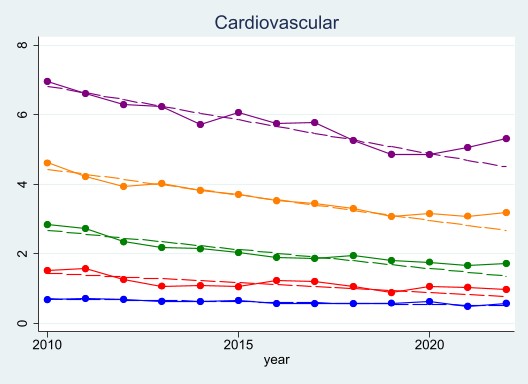

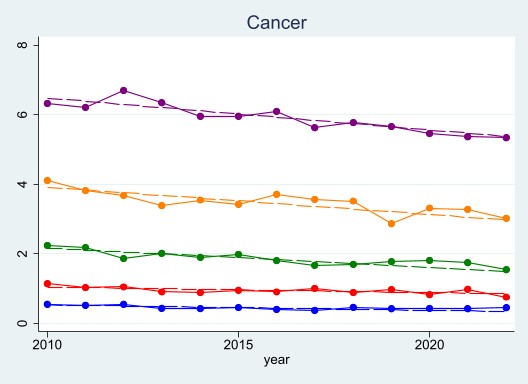


# Fig. S13: Mortality trends among 45-69-year old males in Finland

Notes: The figure shows deaths per 1000 males in Finland, separated by age group: 45-49-year-olds (blue), 50-54-year-olds (red), 55-59-year-olds (green), 60-64-year-olds (orange), and 65-69-year-olds and older (purple). Linear trends were estimated based on 2010–2019.

# Fig. S14: Mortality trends among 45-69-year old females in Finland

Notes: The figure shows deaths per 1000 females in Finland, separated by age group: 45-49-year-olds (blue), 50-54-year-olds (red), 55-59-year-olds (green), 60-64-year-olds (orange), and 65-69-year-olds and older (purple). Linear trends were estimated based on 2010–2019.

# Fig. S15: Mortality trends among males aged 70 and older in Finland

Notes: The figure shows deaths per 1000 males in Finland, separated by age group: 70-74-year-olds (blue), 75-79-year-olds (red), 80-84-year-olds (green), 85-89-year-olds (orange), 90-94-year-olds (purple), and 95-year-olds and older (brown). Linear trends were estimated based on 2010–2019.

# Fig. S16: Mortality trends among females aged 70 and older in Finland

Notes: The figure shows deaths per 1000 females in Finland, separated by age group: 70-74-year-olds (blue), 75-79-year-olds (red), 80-84-year-olds (green), 85-89-year-olds (orange), 90-94-year-olds (purple), and 95-year-olds and older (brown). Linear trends were estimated based on 2010–2019.

# Fig. S17: Mortality trends among 0-39-year old males in Norway

Notes: The figure shows deaths per 1000 males in Norway, separated by age group: 0-19-year-olds (blue), 20-29-year-olds (red), and 30-39-year-olds (green). Linear trends were estimated based on 2010–2019. In some cases, the actual counts may be zero, but the graphs display small positive values due to imputation.

# Fig. S18: Mortality trends among 0-39-year old females in Norway

Notes: The figure shows deaths per 1000 females in Norway, separated by age group: 0-19-year-olds (blue), 20-29-year-olds (red), and 30-39-year-olds (green). Linear trends were estimated based on 2010–2019. In some cases, the actual counts may be zero, but the graphs display small positive values due to imputation.

# Fig. S19: Mortality trends among 40-69-year old males in Norway

Notes: The figure shows deaths per 1000 males in Norway, separated by age group: 40-49-year-olds (blue), 50-59-year-olds (red), and 60-69-year-olds (green). Linear trends were estimated based on 2010–2019. In some cases, the actual counts may be zero, but the graphs display small positive values due to imputation.

# Fig. S20: Mortality trends among 40-69-year old females in Norway

Notes: The figure shows deaths per 1000 males in Norway, separated by age group: 40-49-year-olds (blue), 50-59-year-olds (red), and 60-69-year-olds (green). Linear trends were estimated based on 2010–2019. In some cases, the actual counts may be zero, but the graphs display small positive values due to imputation.

# Fig. S21: Mortality trends among males aged 70 and older in Norway

Notes: The figure shows deaths per 1000 males in Norway, separated by age group: 70-79-year-olds (blue), 80-89-year-olds (red), and 90-year-olds and older (green). Linear trends were estimated based on 2010–2019.

# Fig. S22: Mortality trends among females aged 70 and older in Norway

Notes: The figure shows deaths per 1000 females in Norway, separated by age group: 70-79-year-olds (blue), 80-89-year-olds (red), and 90-year-olds and older (green). Linear trends were estimated based on 2010–2019.

# Fig. S23: Mortality trends among 0-19-year old males in Sweden

Notes: The figure shows deaths per 1000 males in Sweden, separated by age group: 0-4-year-olds (blue), 5-9-year-olds (red), 10-14-year-olds (green), and 15-19-year-olds (orange). Linear trends were estimated based on 2010–2019.

# Fig. S24: Mortality trends among 0-19-year old females in Sweden

Notes: The figure shows deaths per 1000 females in Sweden, separated by age group: 0-4-year-olds (blue), 5-9-year-olds (red), 10-14-year-olds (green), and 15-19-year-olds (orange). Linear trends were estimated based on 2010–2019.

# Fig. S25: Mortality trends among 20-44-year old males in Sweden

Notes: The figure shows deaths per 1000 males in Sweden, separated by age group: 20-24-year-olds (blue), 25-29-year-olds (red), 30-34-year-olds (green), 35-39-year-olds (orange), and 40-44-year-olds and older (purple). Linear trends were estimated based on 2010–2019.

# Fig. S26: Mortality trends among 20-44-year old females in Sweden

Notes: The figure shows deaths per 1000 females in Sweden, separated by age group: 20-24-year-olds (blue), 25-29-year-olds (red), 30-34-year-olds (green), 35-39-year-olds (orange), and 40-44-year-olds and older (purple). Linear trends were estimated based on 2010–2019.

# Fig. S27: Mortality trends among 45-69-year old males in Sweden

Notes: The figure shows deaths per 1000 males in Sweden, separated by age group: 45-49-year-olds (blue), 50-54-year-olds (red), 55-59-year-olds (green), 60-64-year-olds (orange), and 65-69-year-olds and older (purple). Linear trends were estimated based on 2010–2019.

# Fig. S28: Mortality trends among 45-69-year old females in Sweden

Notes: The figure shows deaths per 1000 females in Sweden, separated by age group: 45-49-year-olds (blue), 50-54-year-olds (red), 55-59-year-olds (green), 60-64-year-olds (orange), and 65-69-year-olds and older (purple). Linear trends were estimated based on 2010–2019.

# Fig. S29: Mortality trends among males aged 70 and older in Sweden

Notes: The figure shows deaths per 1000 males in Sweden, separated by age group: 70-74-year-olds (blue), 75-79-year-olds (red), 80-84-year-olds (green), 85-89-year-olds (orange), 90-94-year-olds (purple), and 95-year-olds and older (brown). Linear trends were estimated based on 2010–2019.

# Fig. S30: Mortality trends among females aged 70 and older in Sweden

Notes: The figure shows deaths per 1000 females in Sweden, separated by age group: 70-74-year-olds (blue), 75-79-year-olds (red), 80-84-year-olds (green), 85-89-year-olds (orange), 90-94-year-olds (purple), and 95-year-olds and older (brown). Linear trends were estimated based on 2010–2019.

# Fig. S31: Actual and expected deaths in Denmark

Notes: is actual deaths, is deaths expected based on linear ten-year trends (2010–2019), is deaths expected based on linear five-year trends (2015–2019), and is deaths expected based on log-linear ten-year trends (2010–2019). In the panel with respiratory deaths, is actual deaths also including COVID-19. Expected deaths exhibit non-linear patterns also when based on the linear models due to changes in the sizes of the population strata.

# Fig. S32: Actual and expected deaths in Finland

Notes: is actual deaths, is deaths expected based on linear ten-year trends (2010–2019), is deaths expected based on linear five-year trends (2015–2019), and is deaths expected based on log-linear ten-year trends (2010–2019). In the panel with respiratory deaths, is actual deaths also including COVID-19. Expected deaths exhibit non-linear patterns also when based on the linear models due to changes in the sizes of the population strata.

# Fig. S33: Actual and expected deaths in Norway

Notes: is actual deaths, is deaths expected based on linear ten-year trends (2010–2019), is deaths expected based on linear five-year trends (2015–2019), and is deaths expected based on log-linear ten-year trends (2010–2019). In the panel with respiratory deaths, is actual deaths also including COVID-19. Expected deaths exhibit non-linear patterns also when based on the linear models due to changes in the sizes of the population strata.

# Fig. S34: Actual and expected deaths in Sweden

Notes: is actual deaths, is deaths expected based on linear ten-year trends (2010–2019), is deaths expected based on linear five-year trends (2015–2019), and is deaths expected based on log-linear ten-year trends (2010–2019). In the panel with respiratory deaths, is actual deaths also including COVID-19. Expected deaths exhibit non-linear patterns also when based on the linear models due to changes in the sizes of the population strata.
